# Horizontal gene transfer of *acetyltransferases, invertases* and *chorismate mutases* from different bacteria to diverse recipients

**DOI:** 10.1186/s12862-016-0651-y

**Published:** 2016-04-12

**Authors:** Jason B. Noon, Thomas J. Baum

**Affiliations:** Department of Plant Pathology and Microbiology, Iowa State University, Ames, IA 50011 USA

**Keywords:** Hoplolaimina, Plant-parasitic nematodes, Horizontal gene transfer, Phylogenetics, Model selection analysis, Evolution

## Abstract

**Background:**

Hoplolaimina plant-parasitic nematodes (PPN) are a lineage of animals with many documented cases of horizontal gene transfer (HGT). In a recent study, we reported on three likely HGT candidate genes in the soybean cyst nematode *Heterodera glycines*, all of which encode secreted candidate effectors with putative functions in the host plant. Hg-GLAND1 is a putative GCN5-related N-acetyltransferase (GNAT), Hg-GLAND13 is a putative invertase (INV), and Hg-GLAND16 is a putative chorismate mutase (CM), and blastp searches of the non-redundant database resulted in highest similarity to bacterial sequences. Here, we searched nematode and non-nematode sequence databases to identify all the nematodes possible that contain these three genes, and to formulate hypotheses about when they most likely appeared in the phylum Nematoda. We then performed phylogenetic analyses combined with model selection tests of alternative models of sequence evolution to determine whether these genes were horizontally acquired from bacteria.

**Results:**

Mining of nematode sequence databases determined that *GNATs* appeared in Hoplolaimina PPN late in evolution, while both *INVs* and *CM*s appeared before the radiation of the Hoplolaimina suborder. Also, Hoplolaimina GNATs, INVs and CMs formed well-supported clusters with different rhizosphere bacteria in the phylogenetic trees, and the model selection tests greatly supported models of HGT over descent via common ancestry. Surprisingly, the phylogenetic trees also revealed additional, well-supported clusters of bacterial GNATs, INVs and CMs with diverse eukaryotes and archaea. There were at least eleven and eight well-supported clusters of GNATs and INVs, respectively, from different bacteria with diverse eukaryotes and archaea. Though less frequent, CMs from different bacteria formed supported clusters with multiple different eukaryotes. Moreover, almost all individual clusters containing bacteria and eukaryotes or archaea contained species that inhabit very similar niches.

**Conclusions:**

*GNATs* were horizontally acquired late in Hoplolaimina PPN evolution from bacteria most similar to the saprophytic and plant-pathogenic actinomycetes. *INVs* and *CMs* were horizontally acquired from bacteria most similar to rhizobacteria and *Burkholderia* soil bacteria, respectively, before the radiation of Hoplolaimina. Also, these three gene groups appear to have been frequent subjects of HGT from different bacteria to numerous, diverse lineages of eukaryotes and archaea, which suggests that these genes may confer important evolutionary advantages to many taxa. In the case of Hoplolaimina PPN, this advantage likely was an improved ability to parasitize plants.

**Electronic supplementary material:**

The online version of this article (doi:10.1186/s12862-016-0651-y) contains supplementary material, which is available to authorized users.

## Background

**Fig. 1:**
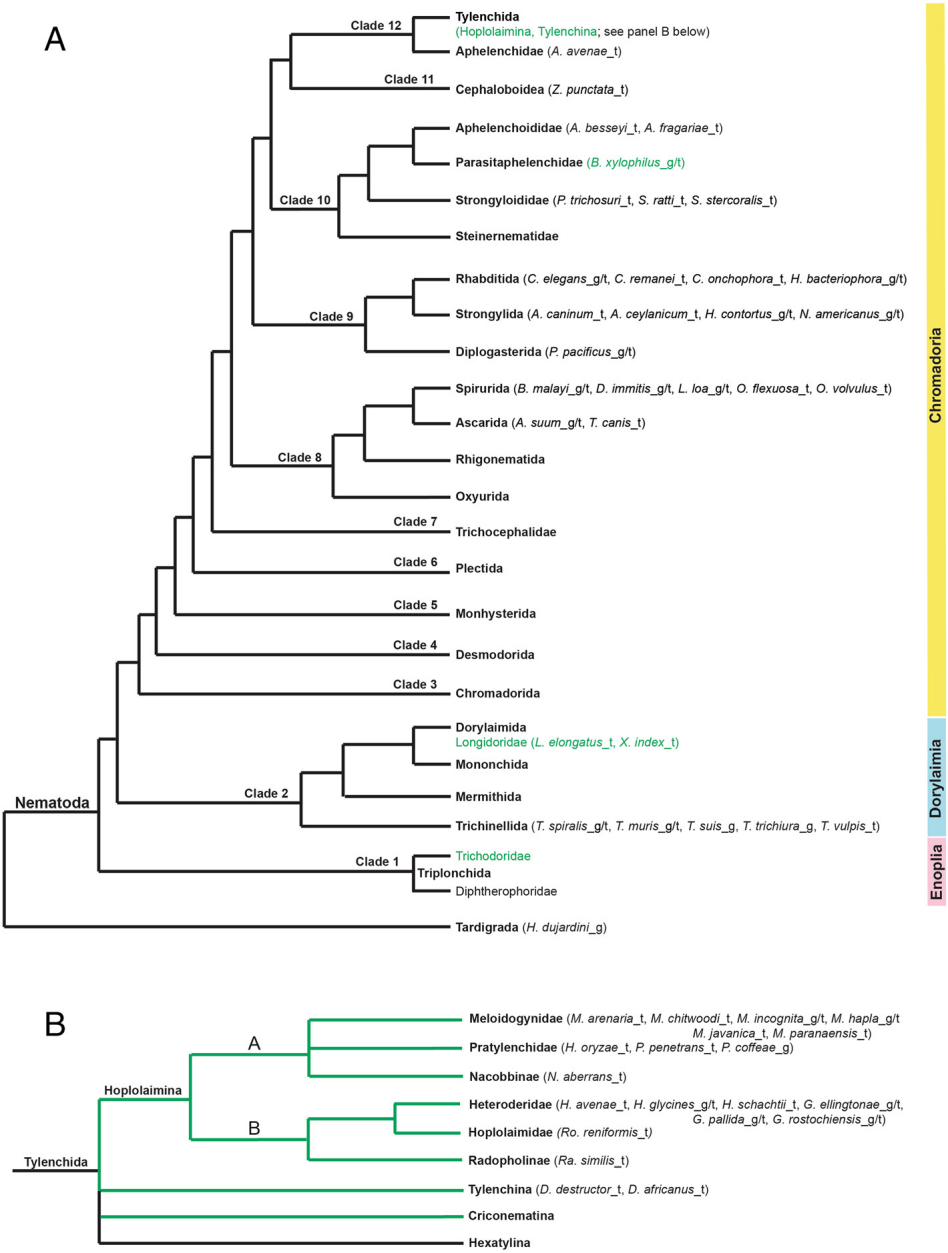
Cladograms of the phylum Nematoda and clade 12 order Tylenchida. Tree topologies of the phylum Nematoda (**a**) and the clade 12 order Tylenchida (**b**) are consistent with that described in [3] and are adapted from [55]. (**a**,**b**) Nematode species whose genomic (_g), transcriptomic (_t), or both genomic and transcriptomic (_g/t) sequences were included in our searches are listed in parentheses at each leaf. Branches that contain PPN species are illustrated in green. These searches included, but were not limited to, information available in nematode sequence databases (see Methods)

Horizontal gene transfer (HGT) is common in bacteria and has recently been documented as an essential evolutionary process for many lineages of eukaryotes (reviewed in [1]). In the phylum Nematoda (Fig. 1a), the plant-parasitic nematodes (PPN) of the suborder Hoplolaimina are among the eukaryotes with the most documented HGT events (reviewed in [2]), especially for HGT from bacterial donors. For example, large suites of genes that encode plant cell wall-modifying proteins were determined to have been acquired in Hoplolaimina PPN via HGT from different bacterial donors [3]. Also, Hoplolaimina PPN were determined to have acquired enzymes for the vitamin B1, B5, B6 and B7 biosynthetic and salvage pathways, also from different bacterial donors [4, 5]. Furthermore, genes encoding invertases (INVs; family 32 glycosyl hydrolases) were recently shown to have been acquired in Hoplolaimina PPN from bacteria, and in the potato cyst nematode *Globodera pallida*, these genes encode functional enzymes that are secreted in the digestive system likely to metabolize host-derived sucrose [6]. Other genes in Hoplolaimina PPN are believed to have bacterial origins, but these hypotheses have not been rigorously tested (reviewed in [2]).

In a recent study, we mined the secretory esophageal gland cells of *Heterodera glycines*, the soybean cyst nematode, to identify new candidate effectors [7]. The esophageal gland cells have been shown to produce secretory proteins with signal peptides that are released into the nematode esophageal lumen and from there, delivered into plant tissues via a specialized hollow mouth spear, the stylet. Candidate nematode effectors are proteins produced specifically in these gland cells with N-terminal signal peptides for secretion into plant hosts (reviewed in [8]). In that study, we identified candidate *H. glycines* effectors Hg-GLAND1 (GenBank: AJR19769.1), Hg-GLAND13 (GenBank: AJR19781.1) and Hg-GLAND16 (GenBank: AJR19784.1) whose mRNAs accumulated specifically in the esophageal gland cells, and whose predicted protein sequences exhibited significant similarities to proteins from different bacteria [7]. All three *Hg-GLAND* genes were identified in a *H. glycines* draft genome and found to contain spliceosomal introns, which indicated that they were not prokaryotic contaminants. The exact roles that these *H. glycines* candidate effectors play in the interaction with the host plant are unknown.

A blastp search of Hg-GLAND1 detected highest sequence similarity to GCN5-related N-acetyltransferases (GNATs) from actinomycetes, predominantly streptomycetes, suggestive of HGT [7]. Also, Hg-GLAND1 contained a predicted GNAT domain (InterPro: IPR000182) [6]. GNATs are the largest known enzyme superfamily functioning in diverse biological processes and are present in bacteria, archaea and eukaryotes [9]. Due to the accumulation of *Hg-GLAND1* mRNAs specifically in the dorsal gland during later parasitic stages (i.e., after the feeding site has completely formed), in combination with the documented defense suppression function of the *Mycobacterium tuberculosis* GNAT effector, we suggested that Hg-GLAND1 might be involved in defense suppression [7].

Interestingly, blastp searches of Hg-GLAND13 detected highest sequence similarity to INVs [7]. In combination with Danchin et al. [6] mentioned above, this finding might suggest that the secretion of an INV effector into the host plant is a relatively new function that evolved outside of the *Globodera* genus, and possibly specifically in *Heterodera* cyst nematodes. We proposed that, in the *H. glycines* feeding site, the Hg-GLAND13 INV may help to increase the metabolic sink potential for compounds derived from photosynthesis, since such a role has been ascribed to INVs from plant hosts of the root-knot nematode *Meloidogyne incognita* [10]. Such a function is supported by the accumulation of the *Hg-GLAND13 INV* mRNAs specifically in dorsal gland during the later parasitic stages [7]. Also, Abad et al. [11] identified two genes in the *Meloidogyne incognita* genome assembly that encode putative INVs that do not contain predicted signal peptides, consistent with the idea that INV effectors might have evolved late in cyst nematode evolution (e.g., specifically in *Heterodera*). Moreover, this finding suggests that, like *G. pallida* INVs [6], these *M. incognita* INVs function within the nematode, but that different from *G. pallida*, they may not be secreted in the digestive system. Furthermore, studies of PPN INVs have determined that these genes were probably acquired in PPN via HGT from bacteria that were most similar to rhizobacteria, nitrogen-fixing symbionts of leguminous plants [6, 11, 12]. Consistent with this conviction, the Hg-GLAND13 INV candidate effector resulted in highest sequence similarity to INVs from *Rhizobium* spp. [7].

Blastp searches of Hg-GLAND16 revealed highest sequence similarity to chorismate mutases (CMs) [7]. CMs are common in bacteria, plants, fungi and apicomplexan parasites, but rare in animals. This is due to the presence of the shikimate pathway, for which CMs convert chorismate into prephenate in the former organisms, and its absence in animals [13]. There are two structural types of CMs: type 1 or AroH class, which is characterized by a trimeric pseudo α/β-barrel structure [14], and type 2 or AroQ class characterized by a dimeric α-helical structure [15]. Interestingly, nematodes do not contain the shikimate pathway, but PPN encode effectors that contain type 2 CM domains [7, 16–20]. Type 1 CM domains have not been reported from nematodes. PPN CMs are largely believed to participate in the suppression of plant defenses [16, 18, 21–23], and to a lesser extent, to induce developmental changes in host plant roots [24, 25]. Also, CMs from *Burkholderia* spp. have been reported most recently as the best match for CM sequences from PPN [22]. Consequently, it has been assumed that CM genes were horizontally acquired in PPN from bacteria [16, 18, 22] (reviewed in [2]). However, these assumptions have been based largely on blast searches and sequence alignments alone, while alternative hypotheses have not been tested (e.g., descent via common ancestry in eukaryotes). Furthermore, while the Hg-GLAND16 candidate effector matched most highly to the previously reported *H. glycines* CMs in blastp analyses and contained a predicted type 2 CM domain (InterPro: IPR002701) [7], Hg-GLAND16 is over four times the size of previously reported *H. glycines* CMs. This observation indicated that the repertoire of CMs in PPN, or at least in *H. glycines*, is more complex than previously appreciated.

Here, we used a combination of bioinformatic, phylogenetic and statistical analyses to rigorously test whether Hg-GLAND1, 13, and 16 were the subject of HGT. We determined how widespread individual gene sequences are within and outside of the phylum Nematoda in order to formulate hypotheses for when HGT would have most likely occurred during nematode evolution. Since all Hg-GLAND1, 13 and 16 homologs that are identified in Hoplolaimina PPN contain the equivalent protein domains mentioned above, we simply refer to them throughout the paper as GNATs, INVs and CMs, respectively. Furthermore, we extensively researched organisms outside of Nematoda to identify likely homologs with the goal to identify putative donor and additional recipient organisms of HGT events. Finally, we tested different models of sequence evolution to explain the presence or absence of gene sequences in different taxa. These analyses confirmed that all three gene groups were acquired from bacteria whose descendants are currently found in the rhizosphere. Surprisingly, we also discovered that homologous ancestral bacterial sequences for two of these gene groups (*GNATs* and *INVs*) were likely the subject of very extensive HGT from highly different bacterial donors to many diverse recipient lineages of eukaryotes and archaea. A similar conclusion can also be drawn for *CMs*, but only to a smaller extent.

## Methods

### Searches of nematode sequence databases

In order to identify putative homologs in other nematodes for *GNATs*, *INVs* and *CMs*, the nucleotide and encoded protein sequences previously identified from *H. glycines* [7] were used as queries for blastn [26] and tblastn searches, respectively, against the transcript contigs, isotigs and genes, as well as to the reads grouped by library databases at Nematode.net [27]. We performed searches against all available nematode clades as well as to Hoplolaimina PPN separately. As outgroups in these searches, we included the genesets from flatworms and *Homo sapiens* at Nematode.net, but as our main outgroup, we included the genomic sequences for the Tardigrade *Hypsibius dujardini* [28]; Tardigrada (water bears) is another basal Ecdysozoa phylum like Nematoda. Also, we performed tblastn and blastp searches against all nucleotide and protein databases available at Nematodes.org, including NEMBASE4 [29], using an E-value threshold of 1E-04 (the online server did not allow 0.001). Furthermore, we performed blastn or tblastn searches against the raw sequence data obtained from the following published or unpublished transcriptome and genome assemblies (E-value thresholds of 0.001): *Heterodera avenae* transcriptome [30], *G. pallida* genome and transcriptome [12], *Globodera rostochiensis* genome and transcriptome (Eves van-den Akker et al., unpublished), *Globodera ellingtonae* genome and transcriptome (Phillips et al., unpublished), *Nacobbus aberrans* transcriptome [31], *Rotylenchulus reniformis* transcriptome (Eves van-den Akker et al., unpublished), *Hirschmaniella oryzae* transcriptome [32], *Pratylenchus coffeae* genome [33], *Pratylenchus penetrans* transcriptome [34], *Ditylenchus destructor* transcriptome ([35]; i.e., 9800 ESTs), *Aphelenchus avenae* transcriptome ([36]; i.e., 5120 ESTs) *Aphelenchoides besseyi* transcriptome [37], *Aphelenchoides fragariae* transcriptome [38], *Bursaphelenchus xylophilus* genome [39], and *Longidorus elongatus* transcriptome (Jones et al., unpublished).

### Searches of NCBI sequences databases

To search for putative, non-nematode homologs of the GNATs, INVs, and CMs, the *H. glycines* homologs were used as queries for blastp [26] searches against the following databases at the National Center for Biotechnology Information (NCBI): non-redundant protein sequences (nr), reference proteins (refseq_protein), patented protein sequences (pat), metagenomic proteins (env_nr), and transcriptome shotgun assembly proteins (tsa_nr). Separate blastp searches were performed specifically against the following taxids for each database: eukaryota taxid 2759, bacteria taxid 2, and archaea taxid 2157. We also searched the expressed sequence tag (EST) database at NCBI using the tblastn algorithm. All searches allowed for 1000 max target sequences and used an Expect (E-value; *E*) threshold of 0.001. Taxonomic classifications of the resulting similar sequences were evaluated using NCBI’s taxonomy reports tool, which implements the taxonomy database at NCBI [40].

In order to maximize our sampling of the above databases for putative homologs of the protein families analyzed, we performed the same searches by using as queries the top bacterial protein sequences that matched most significantly to the respective *H. glycines* proteins. This search greatly increased the quantity of sequences and diversity of taxa that were included in our datasets for more comprehensive phylogenetic analyses.

### Sequence retrieval

All sequences that aligned greater than 50 amino acids within the predicted protein domains of the *H. glycines* proteins (i.e., GNAT, GH32 INV and CM domains), as well as from the best-matching bacterial proteins, with E-values less than 0.001 were kept and inspected for taxonomic classification. All nucleotide sequences obtained from transcriptomic, EST and EST contig databases were translated into protein sequences with the ExPASy translate tool. All genome assembly contigs from Hoplolaimina PPN were subjected to gene model and protein predictions using the self-training eukaryote gene prediction software GeneMark.hmm [41] using the test set from the *C. elegans* genome. For non-nematode taxa, one to ten of the top scoring sequences from each taxonomic group were selected for multiple sequence alignments (MSAs), which allowed us to include a large quantity of sequences from organisms that were distantly related to Hoplolaimina PPN for phylogenetic analyses. No limit was set for the number of Hoplolaimina PPN sequences and all were included in the alignments.

### Multiple sequence alignments

Sequence collections were uploaded into the sequence editor suite of the molecular evolutionary genetics analysis 6 (MEGA6) [42] program. MSAs were performed using the program MUSCLE [43] with default parameters. Sequences that contained substantial gaps with poor alignments to otherwise high quality aligned regions were removed from the analysis in order to maximize the number of informative sites for phylogenetic analysis. Whenever a sequence was removed or edited from an original MSA, the MSA was systemically recalculated. The final MSAs were manually examined using the program Jalview [44].

### Phylogenetic analyses

We performed phylogenetic analyses using bootstrapped Maximum Likelihood (ML). To obtain the most reliable model of amino acid evolution we performed model selection analysis on MSAs using default parameters in the MEGA6 program [42], and the complete results for each gene group analyzed are provided in Additional files 1, 2, 3, 4 and 5. For each protein family analyzed, the evolutionary model that resulted in the lowest Bayesian Information Criterion score was used [42]. Phylogenetic analyses were performed in MEGA6 using ML estimation with 100 bootstrap replications. Reported are the best-scoring ML phylogenetic trees with bootstrap values indicated on the corresponding nodes.

For each protein family analyzed, sequences that resulted in poorly supported clusters, contained relatively long branch lengths, and decreased the confidence of clusters overall within the respective phylogenetic trees were removed. Whenever sequences were removed, MSAs were recalculated, model selection analyses were repeated, and ML phylogenetic trees were re-estimated accordingly. The resulting phylogenetic trees were initially annotated within MEGA6, and then detailed annotations were performed in Adobe Illustrator for visual purposes. The raw phylogenetic trees for each protein family are available in Additional file 6: Figures S1-S5, and include identifications and species names for all sequences used.

### Model selection tests of alternative models of sequence evolution

For each phylogenetic tree presented in the paper, we generated alternative tree topologies from protein MSAs similar to [45] using the Topology Editor tool in MEGA6 [42] in order to rigorously test alternative models of sequence evolution. Taxa were placed into monophyletic groups according to their taxonomic classifications as reported in the taxonomy database at NCBI. Model selection analysis was performed using default parameters on both the original, unconstrained and constrained trees. Reported in Table 1 are the best scoring models of amino acid substitution, the number of parameters associated with the best model, and the Bayesian Information Criterion (BIC) and corrected Akaike Information Criterion (AICc) scores presented as the difference (Δ) from the unconstrained evolutionary models. The raw BIC and AICc scores are provided in Additional file 7. Models that resulted in a difference in BIC and AICc scores of 5 or greater were considered as very strong empirical evidence for the better model [45, 46] (in this work lower scores are better [42]). Each model selection analysis was repeated at least once and we found that the results were identical in all trials.

**Table 1:** Model selection tests of constrained versus unconstrained models of evolution for the candidate Hoplolaimina HGT genes

Evo. Model	Rank	Constraint	Sub. Model	K	∆BIC	∆AICc
*FAM7 GNATs*						
Unconstrained	1	.	LG + G	154	0	0
Constrained 1	8	Euk + Arch	LG + G	154	1175.733	1175.697
Constrained 2	6	Euk	LG + G + I	155	634.763	627.339
Constrained 3	7	CN + Fungi + Mon + Cap	LG + G	154	648.002	647.967
Constrained 4	4	CN + Fungi + Mon	LG + G	154	549.181	549.146
Constrained 5	5	CN + Fungi	LG + G	154	580.907	580.872
Constrained 6	2	CN + L Fungi	LG + G	154	126.561	126.525
Constrained 7	3	CN + E Fungi	LG + G	154	229.615	229.580
*INVs*						
Unconstrained	1	.	WAG + G	232	0	0
Constrained 1	7	Euk + Arch	WAG + G + I	233	2149.761	2140.827
Constrained 2	6	Euk	WAG + G + I	233	1894.372	1885.438
Constrained 3	5	PPN + Insects+ Fungi + P/M/L/C/G/A + Excavates	WAG + G + I	233	1665.100	1656.167
Constrained 4	4	PPN + Insects + Fungi + P/M/L/C/G	WAG + G + I	233	945.151	935.950
Constrained 5	3	PPN + Insects+ Fungi	WAG + G + I	233	822.972	814.039
Constrained 6	2	PPN + Insects	WAG + G + I	233	229.779	220.545
*CMs*						
Unconstrained	1	.	WAG + G + I	89	0	0
Constrained 1	3	Euk	WAG + G + I	89	129.280	129.281
Constrained 2	2	PPN + Insects	WAG + G + I	89	26.109	26.110

*Abbreviations*: *K* number of parameters, *G* Gamma distributed rate variation among amino acid positions, *I* invariant amino acid positions, *Euk* eukaryotes, *Arch* archaea, *CN* cyst nematodes, *PPN* Hoplolaimina plant-parasitic nematodes, Mon *Monosiga brevicollis*, Cap *Capsaspora owczarzaki*, *P/M/L/C/G/A* Plants/Mosses/Lycophytes/*Capsaspora owczarzaki*/Green Algae/*Acanthamoeba castellani*

### %GC content and codon usage comparisons

We calculated %GC content for each coding DNA sequence (cds) using the formula $\left[\frac{\mathrm{G}+\mathrm{C}}{\mathrm{G}+\mathrm{C}+\mathrm{A}+\mathrm{T}}\times 100\right]$. Details for the number of cds included in each distribution, database sources and corresponding cds accession or identification numbers, %GC content for each cds, counts for each %GC content category, placement of confidence intervals, statistics of the distributions, and complete descriptions of how each distribution was generated are provided in Additional file 8. The %GC contents and accession or identification numbers for GNATs, INVs and CMs are provided in Additional file 9. The final distributions shown in Fig. 7 were constructed in JMP Pro version 10.0.2 and were aligned for comparison purposes using Adobe Illustrator. We also analyzed and compared codon usages between the cds of the HGT candidates with codon usage tables from both Hoplolaimina PPN and donor bacteria using the codon adaptation index (CAI) [47]. Complete details for the procedure, accession or identification numbers, codon usage tables used, calculated CAI and expected CAI (E-CAI), and interpretation of the resulting values are provided in Additional file 10.

### Searches for signal peptides, transmembrane regions and protein domains

To search simultaneously for secretion signal peptides and transmembrane (TM) regions, we used a combination of SignalP 4.1 [48], TMHMM 2.0 [49] and Phobius [50]. For SignalP, we used the default D-cutoff values, but implemented both methods—SignalP-TM (input sequences may include TM regions) and SignalP-noTM (input sequences do not include TM regions). For TMHMM and Phobius, we used default parameters. All protein sequences included in our study were inspected for protein domains using a combination of blastp and CD-search [51] at NCBI to search the conserved domains database (CDD) [52], and InterProScan 5 [53] to search the InterPro protein families database [54]. All protein sequences included in our phylogenetic analyses were determined to contain the corresponding protein domains. The InterProScan searches also allowed another round of predictions for signal peptides and TM regions.

## Results and discussion

### Nematode GNAT, INV and CM homologs may be specific to Hoplolaimina

The phylum Nematoda is composed of 12 major clades (Fig. 1a) [55]. Hoplolaimina is found in clade 12 within the order Tylenchida, which in addition to Hoplolaimina contains three additional suborders (Fig. 1b). Tylenchina contains the relatively basal plant-pathogenic Anguinidae, Criconematina contains many plant parasites, and Hexatylina contains the entomopathogenic Sphaerulariidae. Also in clade 12 is the fungal-feeding family Aphelenchidae, and immediately basal to clade 12 is the clade 11 superfamily Cephaloboidea containing strictly bacterial-feeders (Fig. 1a). Furthermore, Hoplolaimina contains strictly plant parasites, and this suborder is subdivided into clades A and B (Fig. 1b). Hoplolaimina clade A contains root-knot (family Meloidogynidae; *Meloidogyne* spp.), lesion (family Pratylenchidae) and false root-knot (family Nacobbinae) nematodes (Fig. 1b). Hoplolaimina clade B contains cyst (family Heteroderidae), reniform (family Hoplolaimidae) and burrowing (subfamily Radopholinae) nematodes (Fig. 1b).

**Fig. 2:**
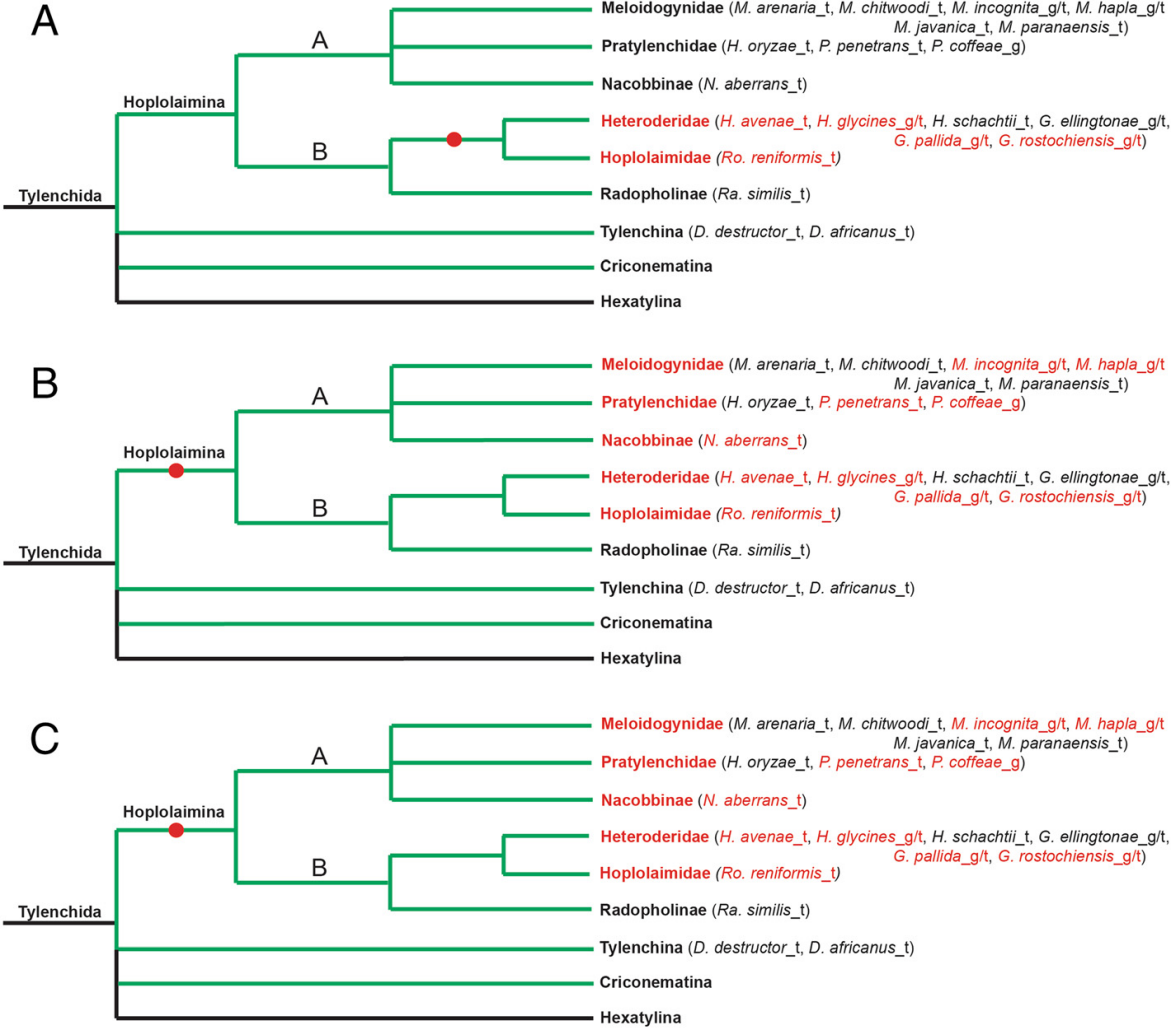
Suspected timing of appearance of *GNATs*, *INVs* and *CMs* in Hoplolaimina PPN. (**a**-**c**) Cladograms are shown as in Fig. 1B. Lineages, and species within, that were found to contain homologs of the HGT genes in question are colored red. The suspected timing of appearance of *GNATs* (**a**), *INVs* (**b**) and *CMs* (**c**) are illustrated with a red circle placed on the appropriate branch. Note that species within a red Hoplolaimina lineage (family or subfamily) that are not colored red does not mean that they do not contain that particular gene, it means that we could not identify that gene in their sequence data, which may be due to insufficient sequence data rather than gene loss. The same goes for the Radopholinae lineage, as *Radopholus similis* was the only species included, which has only limited EST sequences

As a first step in our analyses, we performed a comprehensive search of available nematode genomic and transcriptomic sequences to identify homologs of the three candidate HGT genes in question (i.e., *GNATs*, *INVs* and *CMs*) in parasitic and non-parasitic nematode species other than *H. glycines* (Fig. 1). Our searches included extensive genomic and/or transcriptomic sequences from Nematoda clades 2 and 8–12, and all of the Hoplolaimina (sub)families mentioned above, totaling 51 different nematode species. Two nematode species included only genomic sequences, 30 included only transcriptomic sequences, and 18 included both genomic and transcriptomic sequences. For those nematode species that only included transcriptomic sequences, we cannot rule out the possibility that lack of gene identification is due to lack of gene expression, rather than gene absence entirely. Noteworthy, multiple species within Nematoda clades 2 and 8–10, as well as multiple species within Hoplolaimina clades A and B, included both genomic and transcriptomic sequences (Fig. 1). In these analyses, all three candidate HGT genes were identified to different degrees in Hoplolaimina PPN (Fig. 2), as further described below, but we did not find any significant nematode matches (*E* < 0.001) outside of this suborder. This included the lack of identification within Nematoda clades 2 and 8–11 (Fig. 1), as well as the Tylenchina suborder basal to Hoplolaimina (Figs. 1b and 2); however, the latter only included transcriptomic sequences. Thus, these results suggested that the three candidate HGT genes might only be present within Hoplolaimina, but there is not enough sufficient sequence data available yet throughout Nematoda to be absolutely certain.

Within Hoplolaimina, *GNATs* were completely absent from the three PPN families within clade A, while a single *GNAT* homolog was found in cyst and reniform nematodes within clade B (Fig. 2a). Different from *GNATs*, multiple *INV* (Fig. 2b) and *CM* (Fig. 2c) homologs were identified throughout both Hoplolaimina clades A and B PPN. Thus, these findings indicated that the *GNATs* likely appeared in Hoplolaimina clade B after the divergence from Hoplolaimina clade A, while *INVs* and *CMs* likely appeared before the divergence of Hoplolaimina.

Although it was conceivable that the *GNATs* appeared in Hoplolaimina clade B PPN after divergence from Hoplolaimina clade A, *Radopholus similis* is the only species with sequences to represent the burrowing nematodes, and only has limited transcriptomic sequences (Fig. 2a; 7282 ESTs in GenBank). Thus, it remains possible that *GNATs* could also be present within this Hoplolaimina clade B basal lineage.

Due to insufficient representation of genomic and transcriptomic sequences basal to Hoplolaimina, it was not possible to predict the precise appearance of *INVs* and *CMs* within the distal Nematoda clades (Fig. 1). Though it was conceivable that these two candidate HGT genes appeared before the divergence of Hoplolaimina, we only had limited transcriptomic sequences for the Tylenchina suborder basal to Hoplolaimina (ESTs from *Ditylenchus destructor* and *D. africanus*), and no sequences were available for Criconematina or Hexatylina (Figs. 1b and 2b,c). Our searches did include transcriptomic sequences for the Aphenchidae species *Aphelenchus avenae* immediately basal to Tylenchida in clade 12 (Fig. 1a), but this was limited to just 5,120 ESTs. Furthermore, transcriptomic sequences were only available for a single species within the clade 11 Cephaloboidea lineage (Fig. 1a). On the other hand, sufficient transcriptomic sequences were included for 6 nematode species representing 3 of the 4 main lineages within clade 10, and the facultative plant-parasitic species *Bursaphelenchus xylophilus* included both genomic and transcriptomic sequences, thus providing strong support for the absence of *INVs* and *CMs* from this clade and possibly the more basal Nematoda clades. Thus, it remains possible that *INVs* and *CMs* could be present throughout clades 11 and 12 nematodes, and better sequence representation for these lineages in the future will determine the precise conservation of these candidate HGT genes.

### Hoplolaimina GNATs, INVs and CMs cluster with bacteria in phylogenetic analyses

We had determined that the three candidate HGT genes in question (i.e., *GNATs*, *INVs* and *CMs*) were only present in certain nematode species, and might even be specific to Hoplolaimina PPN. Next, to test the hypothesis that all three candidate HGT genes were horizontally acquired in Hoplolaimina PPN, we performed blastp searches to identify all possible homologs in every NCBI protein sequence database as well as the EST database in order to conduct the most comprehensive phylogenetic analyses possible. If the Hoplolaimina sequences were to cluster with similar bacterial sequences over other similar eukaryotic, or even archaeal sequences, this would support HGT over vertical inheritance. Finally, we used model selection analyses to compare the likelihoods of HGT versus descent via common ancestry in order to provide the most rigorous support for one evolutionary scenario over the other.

For our blastp searches, we used the complete Hoplolaimina GNAT, INV and CM protein sequences as queries, and the protein sequence hits with similarities of *E* < 0.001 to the predicted protein domains were considered as potential homologs, and were thus used in downstream phylogenetic analyses. The majority of protein sequence hits from these analyses were from bacteria. Thus, in order to maximize our sampling of protein sequences from eukaryotes and archaea, in addition to bacteria, we performed separate blastp searches using the bacterial homologs as queries and also considered the resulting non-bacterial protein sequence hits as potential homologs for downstream phylogenetic analyses. For both Hoplolaimina GNATs and INVs, we identified hundreds of potentially homologous sequences covering all three domains of life (eukaryotes, archaea and bacteria), while for CMs, potentially homologous sequences were only found in bacteria and a few other eukaryotes.

**Fig. 3:**
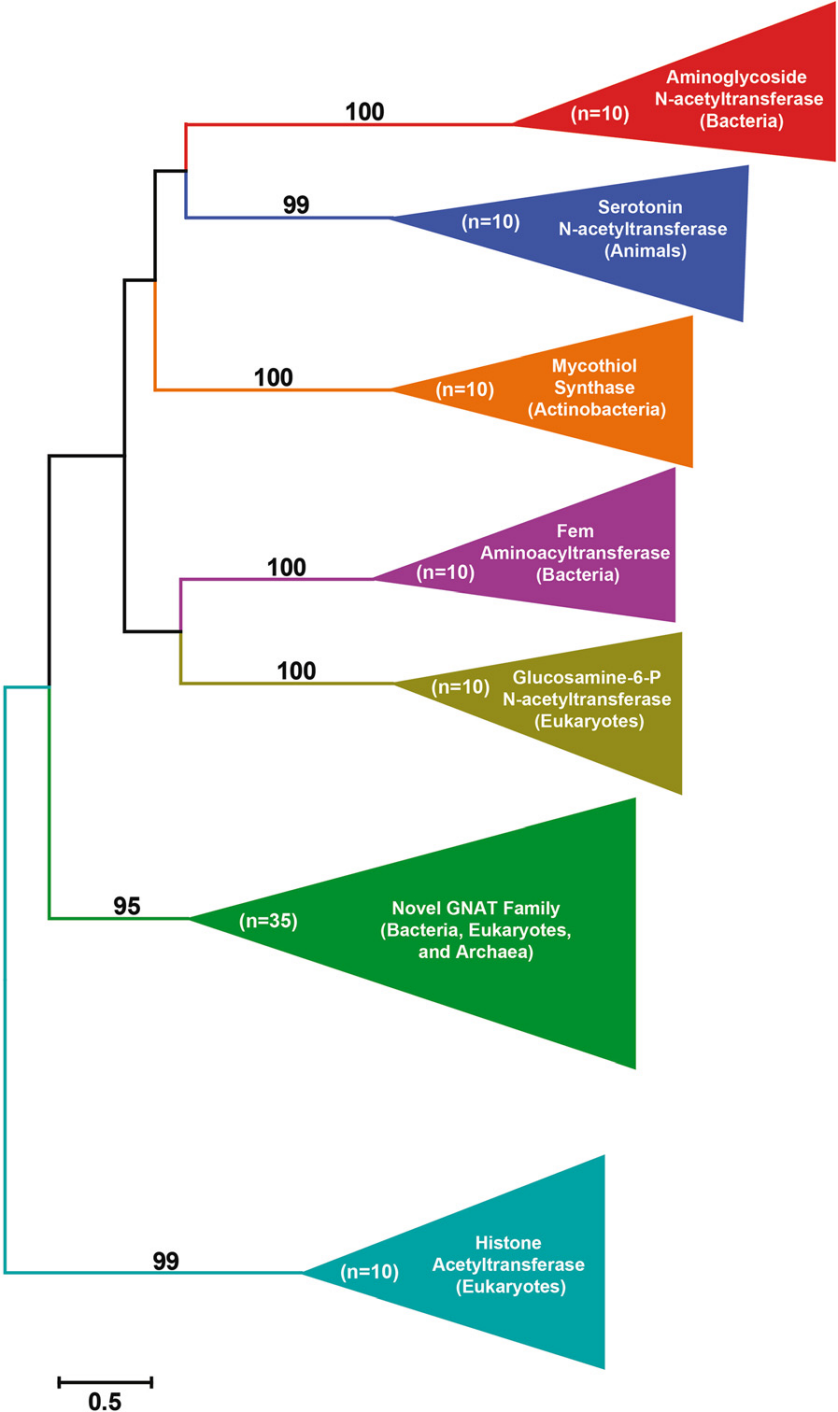
Phylogenetic tree of the GNAT superfamily and newly identified GNATs similar to the Hoplolaimina homologs. Phylogenetic groups containing each GNAT family are collapsed and color-coded with corresponding bootstrap support values indicated at each node. The number of sequences (n) that were used for each GNAT family is indicated within each collapsed phylogenetic group. Organisms that contain each GNAT family are provided in parentheses within each collapsed phylogenetic group. Note that the newly identified GNAT clade with similarity to the Hoplolaimina homologs forms a highly supported monophyletic group with no significant clustering to any other GNAT family, thus indicating a novel GNAT family, which we called Family 7 (FAM7). The raw phylogenetic tree is shown in Additional file 6: Figure S1, and contains all identifiers and species names for all of the sequences that were included in the analysis

We made a particularly interesting discovery when all GNAT sequences discovered by these searches were analyzed. GNATs have been reported to fall into one of the following six families based on sequence, structure and function (although no extensive phylogenetic analyses have been reported to date): bacterial aminoglycoside N-acetyltransferases (NATs), animal serotonin NATs, actinobacterial mycothiol synthases, bacterial Fem aminoacyltransferases, eukaryote glucosamine-6-phosphate NATs, and eukaryote histone acetyltransferases (reviewed in [9]). Thus, before testing the HGT hypothesis, we were interested in determining which GNAT family the Hoplolaimina GNATs belong to. Blastp searches using Hoplolaimina GNATs or their most similar bacterial sequences (i.e., actinomycete GNAT sequences) as queries revealed significant similarities (*E* < 0.001) to protein sequences from other bacteria and archaea, as well as to other eukaryotes. Because no phylogenetic analyses had been reported for GNATs to date, we constructed a ML phylogenetic tree that included the Hoplolaimina GNATs and their blastp hits identified by us along with a large number of known representatives from all six GNAT families. As expected, this analysis showed that all six known GNAT families formed highly supported monophyletic groups (Fig. 3). However, this analysis also resulted in a seventh, highly supported monophyletic group for all Hoplolaimina GNAT sequences along with all bacterial, archaea and other eukaryotic GNAT sequences identified in our blastp searches (Fig. 3, Novel GNAT Family). These findings strongly suggested that Hoplolaimina GNATs and their blastp matches form a novel, seventh GNAT family that has not been described. It can also be speculated that the lack of clustering of this seventh GNAT family to the other six GNAT families suggests that these sequences are not GNATs. However, prediction of GNAT domains in all sequences of the seventh cluster, including all Hoplolaimina GNATs, suggests otherwise, and thus, we refer to the collection of these sequences throughout the rest of the paper as Family 7 (FAM7) GNATs.

**Fig. 4:**
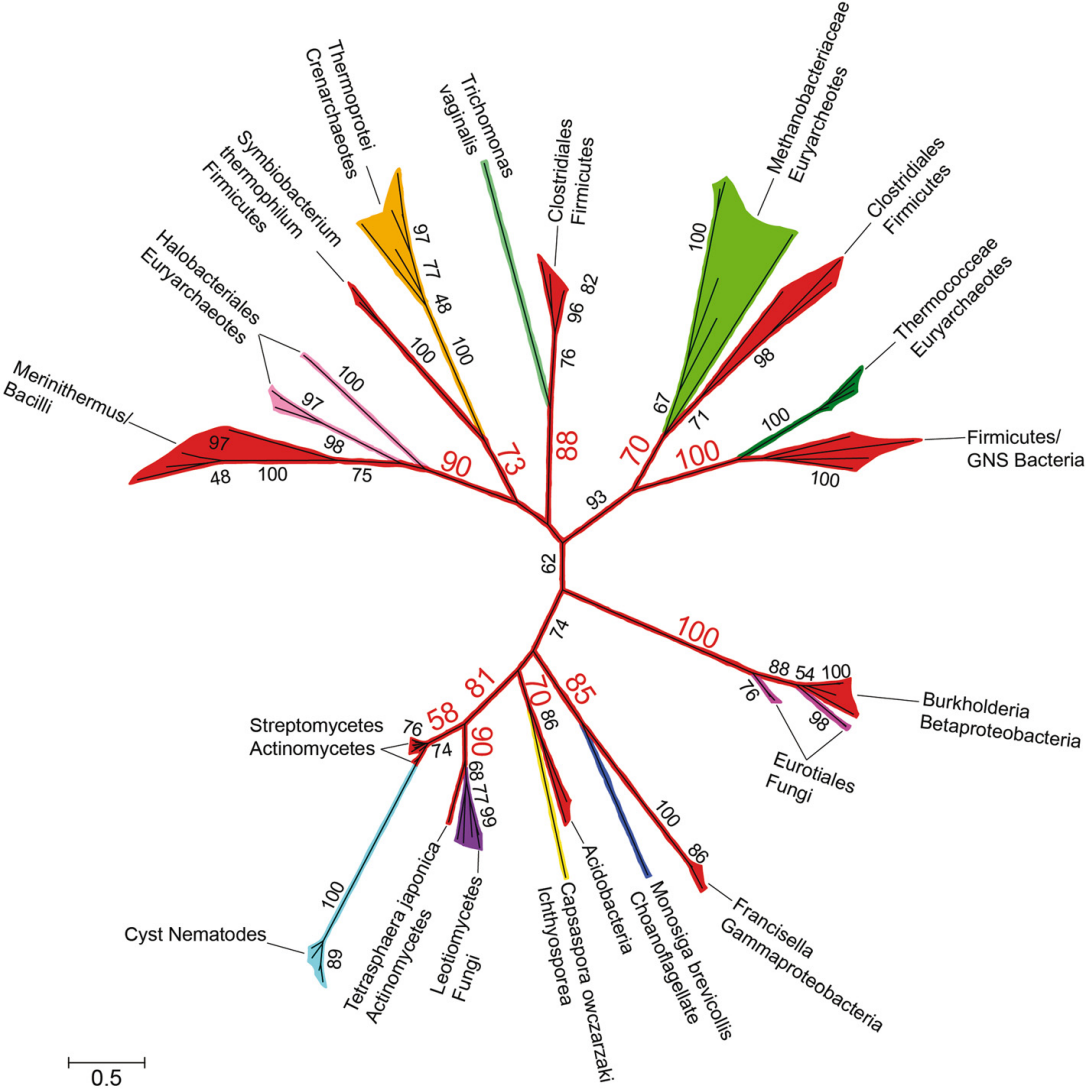
Phylogenetic tree of FAM7 GNATs including the Hoplolaimina homologs. Phylogenetic groups are color-coded according to their taxonomic classifications. Bootstrap support values are indicated at corresponding nodes, and those that support possible HGT events are oversized in red font. Notice a maximum of 10 possible HGT events where eukaryotes and archaea form monophyletic groups with different bacteria, including cyst nematodes with actinomycetes most similar to streptomycetes. The raw phylogenetic tree is shown in Additional file 6: Figure S2, and contains all identifiers and species names for all of the sequences that were included in the analysis

In the ML phylogenetic tree of FAM7 GNATs (Fig. 4), which contained over one hundred sequences, Hoplolaimina clustered with actinomycetes (we included streptomycete sequences since these are the bacterial sequences that are most similar to the Hoplolaimina sequences). Although the bootstrap support for the cluster containing streptomycetes and Hoplolaimina (labeled cyst nematodes in Fig. 4) is not highly supported (bootstrap = 58), the next closest node supporting the larger cluster of cyst nematodes, streptomycetes, the actinomycete *Tetrasphaera japonica* and leotiomycete fungi is well supported (bootstrap = 81). Also, within this cluster *T. japonica* and leotiomycete fungi are in a highly supported cluster (bootstrap = 90), lending additional support for the cluster containing streptomycetes and Hoplolaimina (Fig. 4).

**Fig. 5:**
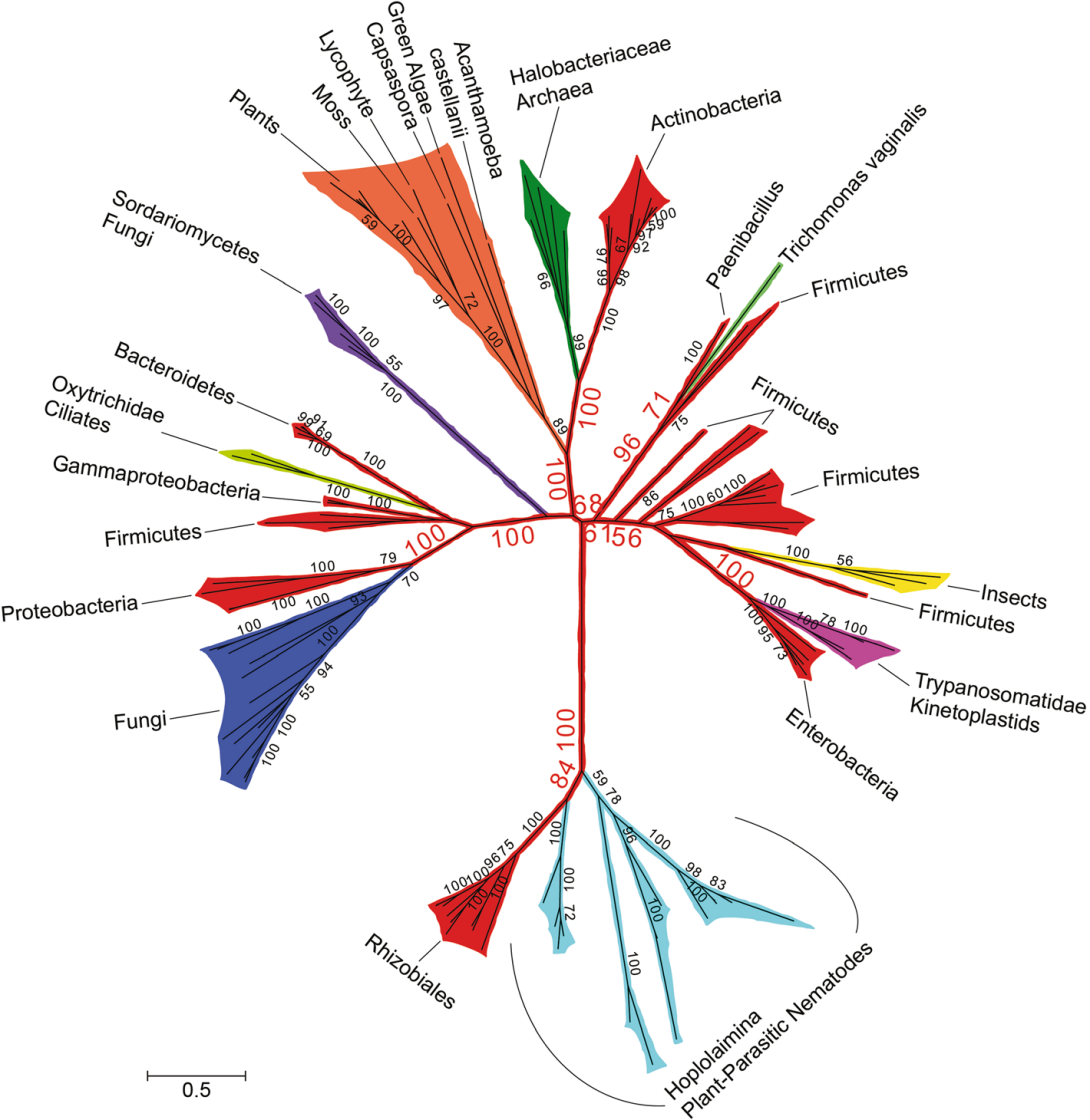
Phylogenetic tree of INVs similar to the Hoplolaimina homologs. Phylogenetic groups are color-coded according to their taxonomic classifications. Bootstrap support values are indicated at corresponding nodes, and those that support possible HGT events are oversized in red font. Notice a maximum of 8 possible HGT events where eukaryotes and archaea form monophyletic groups with different bacteria, including Hoplolaimina PPN with rhizobacteria (order Rhizobiales). The raw phylogenetic tree is shown in Additional file 6: Figure S4, and contains all identifiers and species names for all of the sequences that were included in the analysis

Similarly, Hoplolaimina clustered with rhizobacteria (order Rhizobiales) with very strong support (bootstrap = 100) in the ML phylogenetic tree of INVs (Fig. 5), which also contained over one hundred sequences. These findings are consistent with Danchin et al. [6], and provide additional, rigorous support for this evolutionary relationship.

**Fig. 6:**
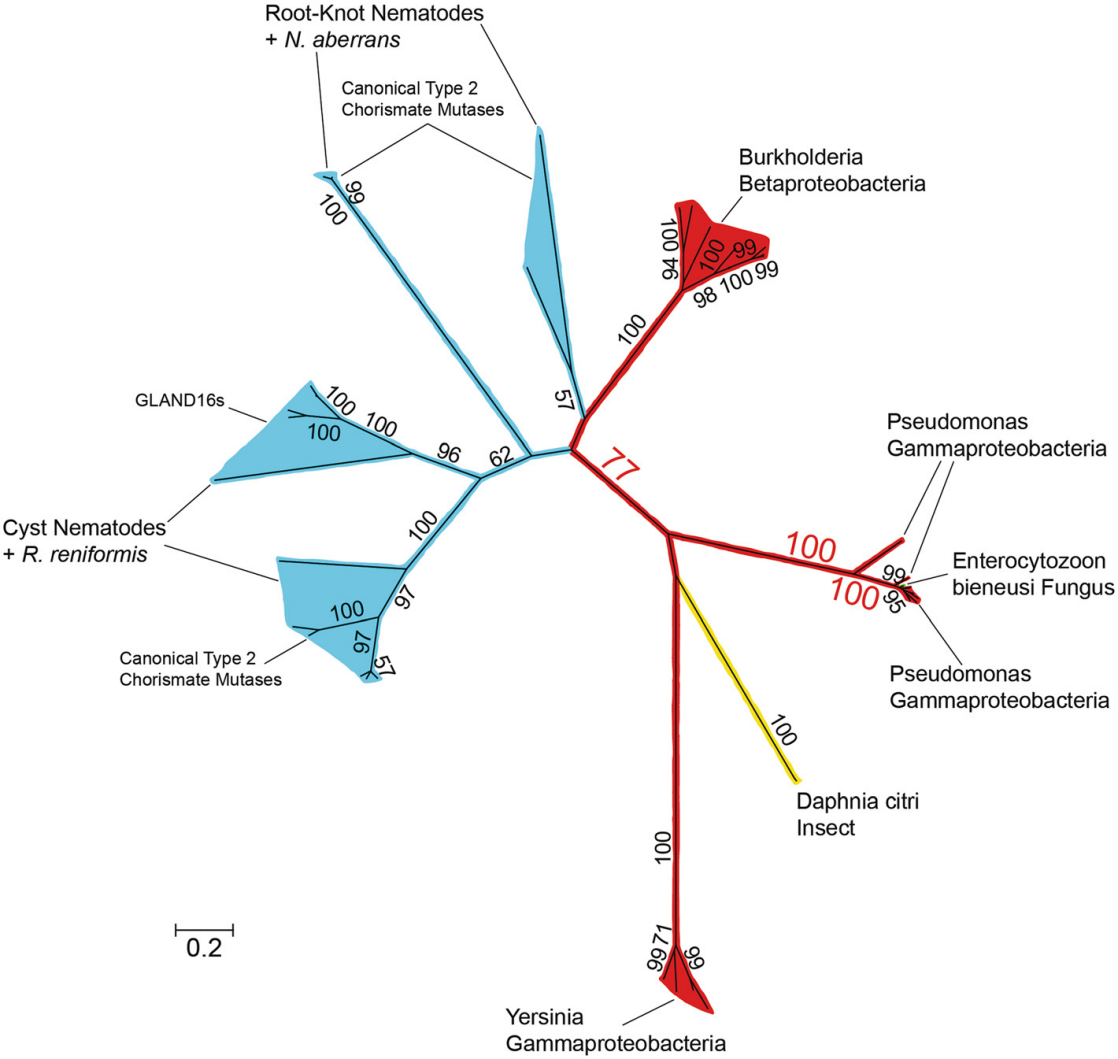
Phylogenetic tree of CMs similar to the Hoplolaimina homologs. Phylogenetic groups are color-coded according to their taxonomic classifications. Bootstrap support values are indicated at corresponding nodes, and those that support possible HGT events are oversized in red font. Notice a supported monophyletic grouping of Hoplolaimina PPN with *Burkholderia* CMs. The raw phylogenetic tree is shown in Additional file 6: Figure S5, and contains all identifiers and species names for all of the sequences that were included in the analysis

Finally, in the ML phylogenetic tree of CMs (Fig. 6), which was much smaller than the FAM7 GNAT and INV phylogenetic trees, but still included all possible homologs that were identified in other eukaryotes from the NCBI sequence databases, Hoplolaimina PPN CMs formed a supported cluster (bootstrap = 77) with *Burkholderia* CMs.

Taken together, these results strongly supported the hypothesis that all three candidate HGT genes were horizontally acquired in Hoplolaimina PPN from bacteria. Importantly, all three suspected bacterial donors are commonly found in the rhizosphere, and thus in the same niche as Hoplolaimina PPN. The latter findings document a physical association between the putative donor and recipient organisms, which further supported our HGT hypothesis.

### *FAM7 GNATs*, *INVs* and *CMs* were horizontally acquired in Hoplolaimina from rhizosphere bacteria

The analyses described above determined that the three groups of nematode effector proteins in question cluster with protein sequences of the suspected donor bacteria in phylogenetic analyses that included all possible homologs that can be found in NCBI protein sequence and EST databases. However, phylogenetic analyses alone are insufficient to document HGT, as descent via common ancestry cannot be completely ruled out using this method. Model selection analysis is a formal method for comparing the likelihoods of different models of sequence evolution [42, 45, 46] such as HGT versus descent via common ancestry and has been used to test the hypothesis of a universal common ancestry of life [45]. In model selection analysis, hypothesized trees, constrained by chosen criteria, are constructed for a given sequence alignment, and models of amino acid substitution and the associated scores [in our case, Bayesian and corrected Akaike Information Criteria (BIC and AICc, respectively)] are calculated [42, 45, 46]. This analysis therefore provides a rigorous method for testing HGT versus descent via common ancestry [45, 46], and thus, we employed this methodology here to test HGT of the candidate Hoplolaimina genes. For all three candidate HGT genes, the unconstrained HGT models consisted of the trees that resulted from our phylogenetic analyses (Figs. 4, 5 and 6). For constrained models that were consistent with descent via common ancestry, Hoplolaimina PPN were grouped with taxa according to known taxonomic classifications (Table 1). For each unconstrained and constrained model of evolution, the rank of score, constraint used (if any), model of amino acid substitution that resulted from the analysis, number of parameters used in each analysis, and the resulting BIC and AICc scores expressed as the difference from the unconstrained model are reported in Table 1.

For each of the three candidate HGT genes in question, the unconstrained and all constrained models of evolution resulted in very similar models of amino acid substitution and number of parameters (Table 1). Since the BIC and AICc scores for each model are weighted by both the likelihood and number of parameters used, the differences in scores observed for each model of evolution represent almost exclusively differences in likelihoods rather than differences in the complexities of each model [45, 46]. Accordingly, the unconstrained HGT models for all three candidate HGT genes scored substantially lower (lower scores are better) than all constrained models of evolution that were consistent with descent via common ancestry (Table 1). Models with even the subtlest constraints placed on the unconstrained HGT models resulted in substantially higher scores. For example, placing the Hoplolaimina FAM7 GNATs with leotiomycete fungi rather than with streptomycete FAM7 GNATs (see Fig. 4), and placing Hoplolaimina CMs with insect rather than with *Burkholderia* CMs (see Fig. 6), resulted in substantially higher scores compared to the unconstrained HGT models (Table 1). These results indicated that the rhizosphere bacteria with which the candidate HGT genes clustered in the phylogenetic analyses (Figs. 4, 5 and 6) are likely modern descendants of the HGT bacterial donors of the *FAM7 GNAT*, *INV* and *CM* genes in Hoplolaimina.

### Evolution of *FAM7 GNATs*, *INVs* and *CMs* in Hoplolaimina following HGT from rhizosphere bacteria

**Fig. 7:**
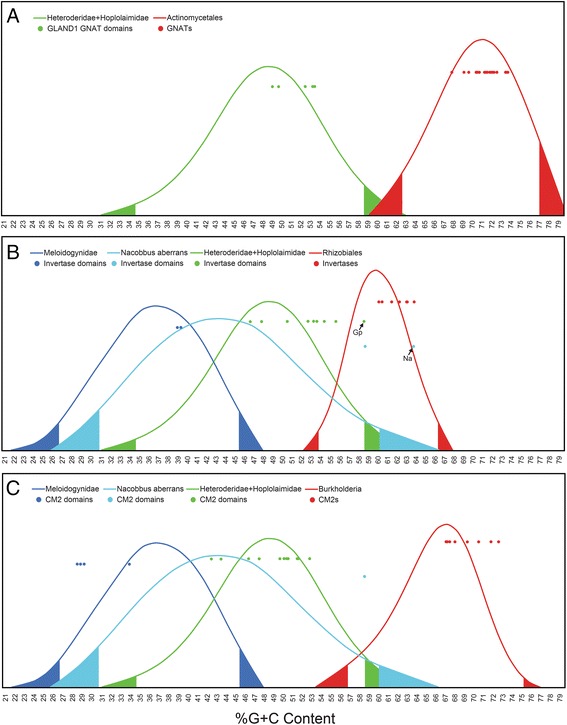
%GC content comparisons of Hoplolaimina HGT genes with distributions constructed from recipients and donors. Distributions of %GC content were constructed using cds from each respective group of Hoplolaimina and donor bacteria listed in each panel. The height of each distribution corresponds to the number of cds at that particular value of %GC content. The x-axis is labeled at the bottom with %GC content. Dots toward the top of each distribution indicate the %GC content for the respective protein domain (transferred form) for the FAM7 GNATs (**a**), INVs (**b**) and CMs (**c**). Dots are included for the donor bacterial genes as reference. Tails on each distribution correspond to the upper and lower limits of two-tailed 95 % confidence intervals. All raw data are provided in Additional files 8 and 9

After determining that the three gene groups in question were horizontally acquired in Hoplolaimina PPN from bacteria most similar to the respective rhizosphere bacteria, we tested whether these genes resembled %GC contents and codon usages similar to the donor or to the recipient genomes. For %GC content, we collected cds for all recipient Hoplolaimina PPN and donor bacteria (Additional file 8) in order to generate distributions of %GC content for each (Fig. 7). %GC contents were calculated for members of each of the three Hoplolaimina HGT gene families (Additional file 9) followed by an evaluation for placement of the calculated %GC contents on each distribution (Fig. 7). Nearly all members evaluated from each of the three HGT gene families resulted in %GC contents similar to the recipient Hoplolaimina genomes (*P* > 0.05; i.e., within the 95 % confidence intervals of each recipient distribution) and significantly different from the donor bacterial genomes (*P* < 0.05; i.e., beyond the 95 % confidence intervals of each donor distribution) (Fig. 7). Only two Hoplolaimina *INVs*, one from *G. pallida* and the other from *N. aberrans*, resulted in %GC contents significantly different (*P* < 0.05) from recipient Hoplolaimina and similar (*P* > 0.05) to donor bacterial genomes (Fig. 7B, *Gp* and *Na*).

For codon usage analyses, we calculated codon adaptation indexes (CAIs) and compared them with the expected CAIs (E-CAIs) [47]. Similar to %GC content, we found that nearly all members of the three HGT gene families used codons that were significantly similar (*P* < 0.05) to Hoplolaimina genomes and different from donor bacterial genomes (Additional file 10). Taken together, these results indicated that subsequent to HGT, all three acquired gene families experienced adaptation to the recipient Hoplolaimina genomes. These findings were consistent with the current paradigm for HGT in PPN that in order for transferred genes to be functional in recipient genomes, they must adapt for efficient transcription and translation [3, 56].

From our above searches for the three HGT genes in nematode genomes and transcriptomes, we found potentially complex patterns of gene duplications in Hoplolaimina following HGT, in particular for the *INVs* and *CMs*. Also, as mentioned above, INVs are understood to be non-secreted in root-knot nematodes [11], to be secreted in the nematode digestive system in the potato cyst nematode *G. pallida* [6], while in the soybean cyst nematode *H. glycines*, they are believed to be secreted effectors (or at least Hg-GLAND13 [7]). Furthermore, previously reported CMs are relatively small proteins that have been documented in all Hoplolaimna PPN with considerable sequence datasets. However, GLAND16 CMs are over four times larger than other CMs [7] and in our above searches were only found in cyst nematodes. Therefore, we were interested in elucidating the complex post-HGT evolution of these gene families in Hoplolaimina. In these analyses, we evaluated the subtrees of the Hoplolaimina recipients and bacterial donors specifically within the ML phylogenetic trees that resulted from our comprehensive phylogenetic analyses, re-evaluated the multiple sequence alignments, and evaluated the protein sequences for predicted protein domains, signal peptides and TM regions. Results from these analyses are detailed in the supplementary text, Additional file 11, and in Additional file 6: Figures S6-S8. In summary, results from these analyses indicated that a *FAM7 GNAT* was acquired from actinomycetes in an ancestor of cyst and reniform nematodes and remains as a single effector gene in each species. Also, *INVs* and *CMs* were acquired from rhizobacteria and *Burkholderia*-related bacteria, respectively, in ancestral Hoplolaimina PPN, and since HGT have experienced multiple duplications with neofunctionalization; some are likely effectors while others are either secreted or TM proteins that function within the nematodes.

### Bacteria were likely HGT hubs of *FAM7 GNATs*, *INVs* and *CMs* to diverse recipients

As mentioned above, from our blastp searches of NCBI protein sequence and EST databases for possible non-nematode homologs of the Hoplolaimina FAM7 GNATs and INVs, we identified numerous possible homologs from all three domains of life (bacteria, archaea and eukaryotes). A total of sixteen different eukayote or archaea lineages were found to contain possible homologs of Hoplolaimina FAM7 GNATs, all of which clustered together in the phylogenetic tree of the GNAT superfamily (Fig. 3). For the INVs, we found a total of nine different eukaryote or archaea lineages that contained possible homologs of the Hoplolaimina INVs. Although to a much smaller extent, we found two additional eukaryotes with possible homologs to Hoplolaimina CMs. Interestingly, all suspected eukaryote and/or archaea homologs of the HGT genes in question matched to different lineages of bacteria in the blastp searches, and most formed well-supported clusters with the different bacteria in the phylogenetic trees (Figs. 4, 5 and 6 and Additional file 6: Figure S5,). There were a total of eleven such examples of different eukaryote and archaea lineages forming well-supported clusters with different bacteria for the FAM7 GNATs (Fig. 4 and Additional file 6: Figure S5), eight such examples for INVs (Fig. 5), and three such examples for CMs (Fig. 6). Moreover, in the NCBI sequence databases, all three HGT genes were found to be present in essentially all bacteria, but only in relatively few eukaryotes and archaea with no indication of common ancestors containing the genes. These findings contradict vertical inheritance being responsible for the presence of any of these genes in these diverse lineages of eukaryotes and archaea. Rather, these findings are best explained by multiple independent HGT events from numerous different bacteria to diverse recipients.

Also important was the finding that many of the suspected donor bacteria (or at least their descendants in cases of more ancient HGTs) for all three HGT genes occupy niches that are very similar to those of the recipient organisms, similar to what we described above for soil dwelling bacterial donors and Hoplolaimina PPN. For example, leotiomycete fungi are commonly found in the soil, and like Hoplolaimina PPN, their suspected *FAM7 GNAT* donors are actinomycete soil bacteria. *Trichomonas vaginalis* and the Clostridiales firmicute *FAM7 GNAT* donor—we mostly identified *Lachnospira multipara*—are found in the human urogenital tract and human intestine, respectively. Also, Trypanosomatids are often found in insects, and the best matches of the Trypanosomatid protein sequences were to the Enterobacteria *Providencia* spp., bacteria that are found in the haeomolymph of some insects. Furthermore, the following bacterial donors to archaea are even more consistent with occupying similar niches: Firstly, Halobacteriales euryarchaeotes consist of extreme halophiles, and one of the most similar bacteria was *Alkalibacillus haloalkaliphilus*, also an extreme halophile. Secondly, Methanobacteriaceae euryarchaeotes consist of extremophiles, and the most similar bacterium was *Dethiobacter alkaliphilus*, also an extremophile. Thirdly, Thermococceae euryarcheatoes are extreme thermophiles, and the most similar bacteria were *Coprothermobacter proteolyticus* and *Thermotoga hypogea*, both extreme thermophiles. Lastly, Thermoprotei crenarchaeotes are also extreme thermophiles, and among the most similar bacteria were *Thermobaculum terrenum* and *Symbiobacterium thermophilum*, again also extreme thermophiles. Taken together, these findings indicated that the majority of all donors and recipients of the HGT genes in question occupy similar niches, which further strengthens the conclusion of numerous, independent HGTs.

## Conclusions

In this study, using a combination of sequence database mining, phylogenetic analyses and tests of alternative models of sequence evolution, we have determined that three gene families in Hoplolaimina PPN were acquired via HGT from different rhizosphere bacteria. These three gene families are the *GLAND1s* (which encode proteins that were determined to be part of a novel family of GNATs which we called FAM7), *INVs* and *CMs*. Some of the homologs from each HGT gene family have evolved into *bona fide* or candidate effectors subsequent to HGT. A *FAM7 GNAT* was acquired in the Hoplolaimina clade B lineage from actinomycetes most similar to streptomycetes and presently encodes the GLAND1 candidate effector in cyst and reniform nematodes. Similarly, *INV* and *CM* genes were acquired in Hoplolaimina from rhizobacteria and *Burkholderia*-related bacteria, respectively, but before the radiation of the suborder. Subsequent to HGT, the acquired *INV* and *CM* genes appear to have experienced complex duplications with neofunctionalization (e.g., some homologs presently encode candidate or *bona fide* effectors, and some encode secreted and TM proteins likely functioning within the nematodes).

Remarkably, we also found that *FAM7 GNATs*, *INVs*, and to lesser extent *CMs*, were likely subjects of numerous HGTs from bacteria to diverse recipients, including both eukaryotes and archaea for the former two genes. The suspected donors for nearly all HGTs occupy very similar niches as the recipient organisms, thus strengthening the conclusion of numerous possible HGTs. These findings indicate that bacteria likely served as hubs for HGT of these three genes to diverse recipients, and demonstrate their likely importance for not just Hoplolaimina PPN, but for many diverse taxa.

### Ethics

Not applicable

### Consent to publish

Not applicable

### Availability of data and materials

The data sets supporting the results of this article are included within the article (and its additional files). Also, the raw phylogenetic data can be found in the Dryad database (doi:10.5061/dryad.pb68n).

## Additional files

Additional file 1: Complete model selection test results for phylogenetic analysis of the GNAT superfamily. (PDF 56 mb) Additional file 2: Complete model selection test results for phylogenetic analysis of the FAM7 GNATs excluding poorly clustered taxa. (XLSX 98 kb) Additional file 3: Complete model selection test results for phylogenetic analysis of the remaining, initially poorly clustered FAM7 GNATs. (XLSX 35 kb) Additional file 4: Complete model selection test results for phylogenetic analysis of the INVs. (XLSX 84 kb) Additional file 5: Complete model selection test results for phylogenetic analysis of the CMs. (PDF 123 kb) Additional file 6: Supplementary figures and legends. (XLS 518 kb) Additional file 7: Raw scores from model selection analyses of alternative models of sequence evolution. (XLS 519 kb) Additional file 8: Raw data from comparisons of %GC content. (XLS 519 kb) Additional file 9: cds from Hoplolaimina and donor bacteria selected for comparisons of %GC content with the respective distributions. (XLS 519 kb) Additional file 10: Codon usage analyses of FAM7 GNAT, INV and CM domains from Hoplolaimina. (XLS 519 kb) Additional file 11: Supplementary text. Evolution of Hoplolaimina HGT genes following transfer from rhizosphere bacteria. (XLSX 9 kb)

## Abbreviations

AICc: corrected Akaike Information Criterion
BIC: Bayesian Information Criterion
CAI: codon adaptation index
CM: chorismate mutase
*E*: E-value
E-CAI: expected codon adaptation index
EST: expressed sequence tag
FAM7 GNAT: family 7 GNAT
GNAT: GCN5-related N-acetyltransferase
HGT: horizontal gene transfer
INV: invertase
ML: Maximum Likelihood
MSA: multiple sequence alignment
NCBI: National Center for Biotechnology Information
PPN: plant-parasitic nematodes
