# Supplementary material for: Horizontal gene transfer of acetyltransferases, invertases and chorismate mutases from different bacteria to diverse recipients
Source: BMC Evol Biol. 2016 Apr 12;16:74. doi: 10.1186/s12862-016-0651-y (PMC4828791; doi:10.1186/s12862-016-0651-y)
Supplement: Additional file 1: — Complete model selection test results for phylogenetic analysis of the GNAT superfamily. (PDF 56 mb) [file 12862_2016_651_MOESM1_ESM.pdf]

Horizontal gene transfer of *acetyltransferases, invertases* and *chorismate mutases* from different bacteria to diverse recipients

Jason B. Noon[1] and Thomas J. Baum[1]*

[1]Department of Plant Pathology and Microbiology, Iowa State University Ames, IA 50011, U.S.A.

Email addresses: JBN, jnoon15@iastate.edu; TJB, tbaum@iastate.edu

*Corresponding author

Supplementary Figure Legends

Figure S1. Raw Maximum Likelihood phylogenetic tree of the GCN5-related N-acetyltransferase (GNAT) superfamily and newly identified GNATs similar to Hoplolaimina homologs. Branches for each GNAT family are color-coded accordingly. Bootstrap support values are indicated at corresponding nodes, and those that support monophyly of each GNAT family are oversized in red font. Names and organisms found to contain each GNAT family are indicated to the right with brackets around the respective leaves. Sequence identifications are provided in parentheses at each leaf.

Figure S2. Detailed Maximum Likelihood phylogenetic tree of the FAM7 GNATs including Hoplolaimina homologs. Branches for each phylogenetic group are color-coded according to their taxonomy. Bootstrap support values are indicated at corresponding nodes, and those that support possible horizontal gene transfer (HGT) events are oversized in red font. Organism names are indicated to the right with brackets around the respective leaves. Sequence identifications are provided in parentheses at each leaf.

Figure S3. Detailed Maximum Likelihood phylogenetic tree of INVs similar to Hoplolaimina homologs. Branches for each phylogenetic group are color-coded according to their taxonomy. Bootstrap support values are indicated at corresponding nodes, and those that support possible horizontal gene transfer (HGT) events are oversized in red font. Organism names are indicated to the right with brackets around the respective leaves. Sequence identifications are provided in parentheses at each leaf.

Figure S4. Detailed Maximum Likelihood phylogenetic tree of CMs similar to Hoplolaimina homologs. Branches for each phylogenetic group are color-coded according to their taxonomy. Bootstrap support values are indicated at corresponding nodes, and those that support possible horizontal gene transfer (HGT) events are oversized in red font. Organism names are indicated to the right with brackets around the respective leaves. Sequence identifications are provided in parentheses at each leaf. Note that the *Nacobbus aberrans* leaf is colored red to indicate its possible neofunctionalization from the other Hoplolaimina homologs (see supplementary text, Additional file 5).

Figure S5. Detailed Maximum Likelihood phylogenetic tree of initially poorly clustered FAM7 GNATs. Branches for each phylogenetic group are color-coded according to their taxonomy. Bootstrap support values are indicated at corresponding nodes, and those that support possible horizontal gene transfer (HGT) events are oversized in red font. Organism names are indicated to the right with brackets around the respective leaves. Sequence identifications are provided in parentheses at each leaf.

Figure S6. Sequence alignment of Hoplolaimina GLAND1 (FAM7 GNAT) proteins. Purple shading illustrates conserved amino acids. A consensus sequence is provided below the alignment. Amino acid positions are indicated to the left and right of each sequence in each row. A green bracket is drawn around the N-terminal signal peptides and a red bracket around the GNAT regions. Notice the extent of conserved amino acids within the GNAT regions, as opposed to the minimal conservation observed outside of the GNAT regions. Hg=*Heterodera glycines*, Ha=*Heterodera avenae*, Gp=*Globodera pallida*, Gr=*Globodera rostochiensis*, *Rr=Rotylenchulus reniformis*.

Figure S7. Evolution of INVs in Hoplolaimina following horizontal gene transfer (HGT) from bacteria most similar to rhizobacteria. (A) Subtree of the cluster containing Hoplolaimina plant parasitic nematodes (PPN) and rhizobacteria (Rhizobiales) calculated from the raw phylogenetic tree in figure S3, Additional file 1. Hoplolaimina PPN branches are colored green and rhizobacteria branches are colored red. Bootstrap support values are indicated at corresponding nodes. Sequence identifications are provided in parentheses at each leaf. Supported clusters are indicated at corresponding nodes with oversized, black bold italicized font. (B) Schematic diagrams of all identified INVs in Hoplolaimina PPN, also listed in panel A. Probable N-terminal signal peptides (SP) are illustrated in green. Regions predicted for transmembrane (TM) domains, with or without an overlapping predicted SP, are illustrated in gray. INV domains (GH32; glycoside hydrolase family 32) are illustrated in red. Protein schematics are drawn according to the scale provided at the bottom. Hg=*Heterodera glycines*, Ha=*Heterodera avenae*, Gr=*Globodera rostochiensis*, Gp=*Globodera pallida*, Rr=*Rotylenchulus reniformis*, Na=*Nacobbus aberrans*, Mi=*Meloidogyne incognita*.

Figure S8. Evolution of CMs in Hoplolaimina following horizontal gene transfer (HGT) from *Burkholderia*-related bacteria. (A) Subtree of the cluster containing Hoplolaimina plant parasitic nematodes (PPN) and *Burkholderia* spp. calculated from the raw phylogenetic tree in figure S4, Additional file 1. Hoplolaimina PPN branches are colored green and *Burkholderia* branches are colored red. Bootstrap support values are indicated at corresponding nodes. Sequence identifications are provided in parentheses at each leaf. Supported clusters are indicated at corresponding nodes with oversized, black bold italicized font. (B) Schematic diagrams of all identified CMs in Hoplolaimina PPN, also listed in panel A. N-terminal signal peptides (SP) are illustrated in green. Transmembrane (TM) regions are illustrated in gray. CM type 2 domains (CM_2s) are illustrated in purple. Proteins with incomplete C-terminal ends are illustrated with dots (…). Protein schematics are drawn according to the scale provided at the bottom. Hoplolaimina PPN groups for all proteins are indicated to the left. Hg=*Heterodera glycines*, Ha=*Heterodera*

avenae, Hs=*Heterodera schachtii*, Gr=*Globodera rostochiensis*, Gp=*Globodera pallida*, Gt=*Globodera tabacum*, Ge=*Globodera ellingtonae*, Rr=*Rotylenchulus reniformis*, Na=*Nacobbus aberrans*, Mi=*Meloidogyne incognita*, Ma=*Meloidogyne artiellia*, Mj=*Meloidogyne javanica*.

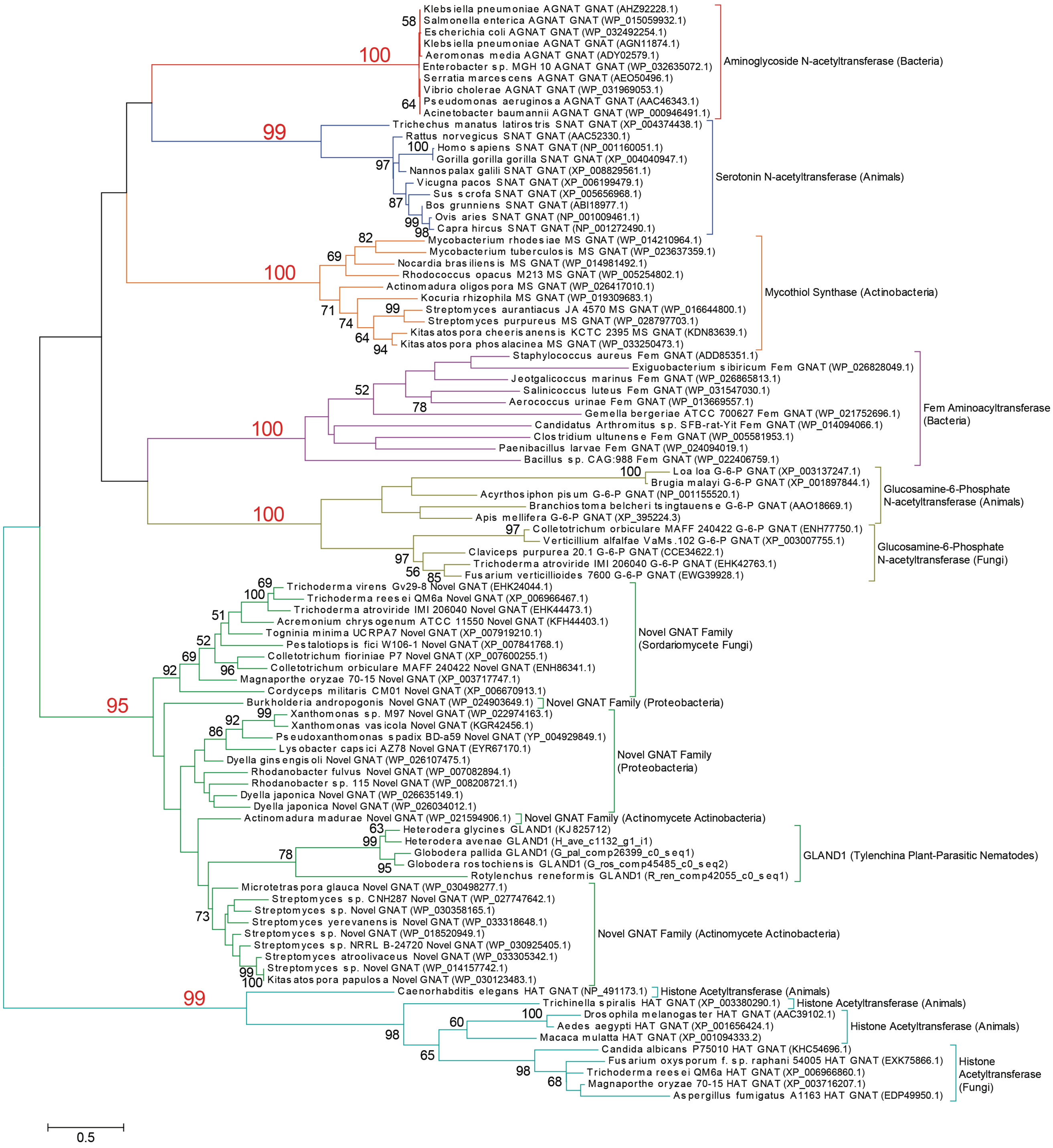
Klebsiella pneumoniae AGNAT GNAT (AHZ92228.1)
Salmonella enterica AGNAT GNAT (WP_015059932.1)
Escherichia coli AGNAT GNAT (WP_032492254.1)
Klebsiella pneumoniae AGNAT GNAT (AGN11874.1)
Aeromonas media AGNAT GNAT (ADY02579.1)
Enterobacter sp. MGH 10 AGNAT GNAT (WP_032635072.1)
Serratia marcescens AGNAT GNAT (AEO50496.1)
Vibrio cholerae AGNAT GNAT (WP_031969053.1)
Pseudomonas aeruginosa AGNAT GNAT (AAC46343.1)
Acinetobacter baumannii AGNAT GNAT (WP_000946491.1)
Aminoglycoside N-acetyltransferase (Bacteria)
Trichechus manatus latirostris SNAT GNAT (XP_004374438.1)
Rattus norvegicus SNAT GNAT (AAC52330.1)
Homo sapiens SNAT GNAT (NP_001160051.1)
Gorilla gorilla gorilla SNAT GNAT (XP_004040947.1)
Nannospalax galili SNAT GNAT (XP_008829561.1)
Vicugna pacos SNAT GNAT (XP_006199479.1)
Sus scrofa SNAT GNAT (XP_005656968.1)
Bos grunniens SNAT GNAT (ABI18977.1)
Ovis aries SNAT GNAT (NP_001009461.1)
Capra hircus SNAT GNAT (NP_001272490.1)
Serotonin N-acetyltransferase (Animals)
Mycobacterium rhodesiae MS GNAT (WP_014210964.1)
Mycobacterium tuberculosis MS GNAT (WP_023637359.1)
Nocardia brasiliensis MS GNAT (WP_014981492.1)
Rhodococcus opacus M213 MS GNAT (WP_005254802.1)
Actinomadura oligospora MS GNAT (WP_026417010.1)
Kocuria rhizophila MS GNAT (WP_019309683.1)
Streptomyces aurantiacus JA 4570 MS GNAT (WP_016644800.1)
Streptomyces purpureus MS GNAT (WP_028797703.1)
Kitasatospora cheerisanensis KCTC 2395 MS GNAT (KDN83639.1)
Kitasatospora phosalacinea MS GNAT (WP_033250473.1)
Mycothiol Synthase (Actinobacteria)
Staphylococcus aureus Fem GNAT (ADD85351.1)
Exiguobacterium sibiricum Fem GNAT (WP_026828049.1)
Jeotgalicoccus marinus Fem GNAT (WP_026865813.1)
Salinicoccus luteus Fem GNAT (WP_031547030.1)
Aerococcus urinae Fem GNAT (WP_013669557.1)
Gemella bergeriae ATCC 700627 Fem GNAT (WP_021752696.1)
Candidatus Arthromitus sp. SFB-rat-Yit Fem GNAT (WP_014094066.1)
Clostridium ultunense Fem GNAT (WP_005581953.1)
Paenibacillus larvae Fem GNAT (WP_024094019.1)
Bacillus sp. CAG:988 Fem GNAT (WP_022406759.1)
Fem Aminoacyltransferase (Bacteria)
Loa loa G-6-P GNAT (XP_003137247.1)
Brugia malayi G-6-P GNAT (XP_001897844.1)
Acyrthosiphon pisum G-6-P GNAT (NP_001155520.1)
Branchiostoma belcheri tsingtauense G-6-P GNAT (AAO18669.1)
Apis mellifera G-6-P GNAT (XP_395224.3)
Glucosamine-6-Phosphate N-acetyltransferase (Animals)
Colletotrichum orbiculare MAFF 240422 G-6-P GNAT (ENH77750.1)
Verticillium alfalfae VaMs.102 G-6-P GNAT (XP_003007755.1)
Claviceps purpurea 20.1 G-6-P GNAT (CCE34622.1)
Trichoderma atroviride IMI 206040 G-6-P GNAT (EHK42763.1)
Fusarium verticillioides 7600 G-6-P GNAT (EWG39928.1)
Glucosamine-6-Phosphate N-acetyltransferase (Fungi)
Trichoderma virens Gv29-8 Novel GNAT (EHK24044.1)
Trichoderma reesei QM6a Novel GNAT (XP_006966467.1)
Trichoderma atroviride IMI 206040 Novel GNAT (EHK44473.1)
Acremonium chrysogenum ATCC 11550 Novel GNAT (KFH44403.1)
Togninia minima UCRPA7 Novel GNAT (XP_007919210.1)
Pestalotiopsis fici W106-1 Novel GNAT (XP_007841768.1)
Colletotrichum fioriniae P7 Novel GNAT (XP_007600255.1)
Colletotrichum orbiculare MAFF 240422 Novel GNAT (ENH86341.1)
Magnaporthe oryzae 70-15 Novel GNAT (XP_003717747.1)
Cordyceps militaris CM01 Novel GNAT (XP_006670913.1)
Novel GNAT Family (Sordariomycete Fungi)
Burkholderia andropogonis Novel GNAT (WP_024903649.1)
Novel GNAT Family (Proteobacteria)
Xanthomonas sp. M97 Novel GNAT (WP_022974163.1)
Xanthomonas vasicola Novel GNAT (KGR42456.1)
Pseudoxanthomonas spadix BD-a59 Novel GNAT (YP_004929849.1)
Lysobacter capsici AZ78 Novel GNAT (EYR67170.1)
Dyella ginsengisoli Novel GNAT (WP_026107475.1)
Rhodanobacter fulvus Novel GNAT (WP_007082894.1)
Rhodanobacter sp. 115 Novel GNAT (WP_008208721.1)
Dyella japonica Novel GNAT (WP_026635149.1)
Dyella japonica Novel GNAT (WP_026034012.1)
Novel GNAT Family (Proteobacteria)
Actinomadura madurae Novel GNAT (WP_021594906.1)
Novel GNAT Family (Actinomycete Actinobacteria)
Heterodera glycines GLAND1 (KJ825712)
Heterodera avenae GLAND1 (H_ave_c1132_g1_i1)
Globodera pallida GLAND1 (G_pal_comp26399_c0_seq1)
Globodera rostochiensis GLAND1 (G_ros_comp45485_c0_seq2)
Rotylenchus reneformis GLAND1 (R_ren_comp42055_c0_seq1)
GLAND1 (Tylenchina Plant-Parasitic Nematodes)
Microtetraspora glauca Novel GNAT (WP_030498277.1)
Streptomyces sp. CNH287 Novel GNAT (WP_027747642.1)
Streptomyces sp. Novel GNAT (WP_030358165.1)
Streptomyces yerevanensis Novel GNAT (WP_033318648.1)
Streptomyces sp. Novel GNAT (WP_018520949.1)
Streptomyces sp. NRRL B-24720 Novel GNAT (WP_030925405.1)
Streptomyces atroolivaceus Novel GNAT (WP_033305342.1)
Streptomyces sp. Novel GNAT (WP_014157742.1)
Kitasatospora papulosa Novel GNAT (WP_030123483.1)
Novel GNAT Family (Actinomycete Actinobacteria)
Caenorhabditis elegans HAT GNAT (NP_491173.1)
Histone Acetyltransferase (Animals)
Trichinella spiralis HAT GNAT (XP_003380290.1)
Histone Acetyltransferase (Animals)
Drosophila melanogaster HAT GNAT (AAC39102.1)
Aedes aegypti HAT GNAT (XP_001656424.1)
Macaca mulatta HAT GNAT (XP_001094333.2)
Histone Acetyltransferase (Animals)
Candida albicans P75010 HAT GNAT (KHC54696.1)
Fusarium oxysporum f. sp. raphani 54005 HAT GNAT (EXK75866.1)
Trichoderma reesei QM6a HAT GNAT (XP_006966860.1)
Magnaporthe oryzae 70-15 HAT GNAT (XP_003716207.1)
Aspergillus fumigatus A1163 HAT GNAT (EDP49950.1)
Histone Acetyltransferase (Fungi)
0.5

Figure S1

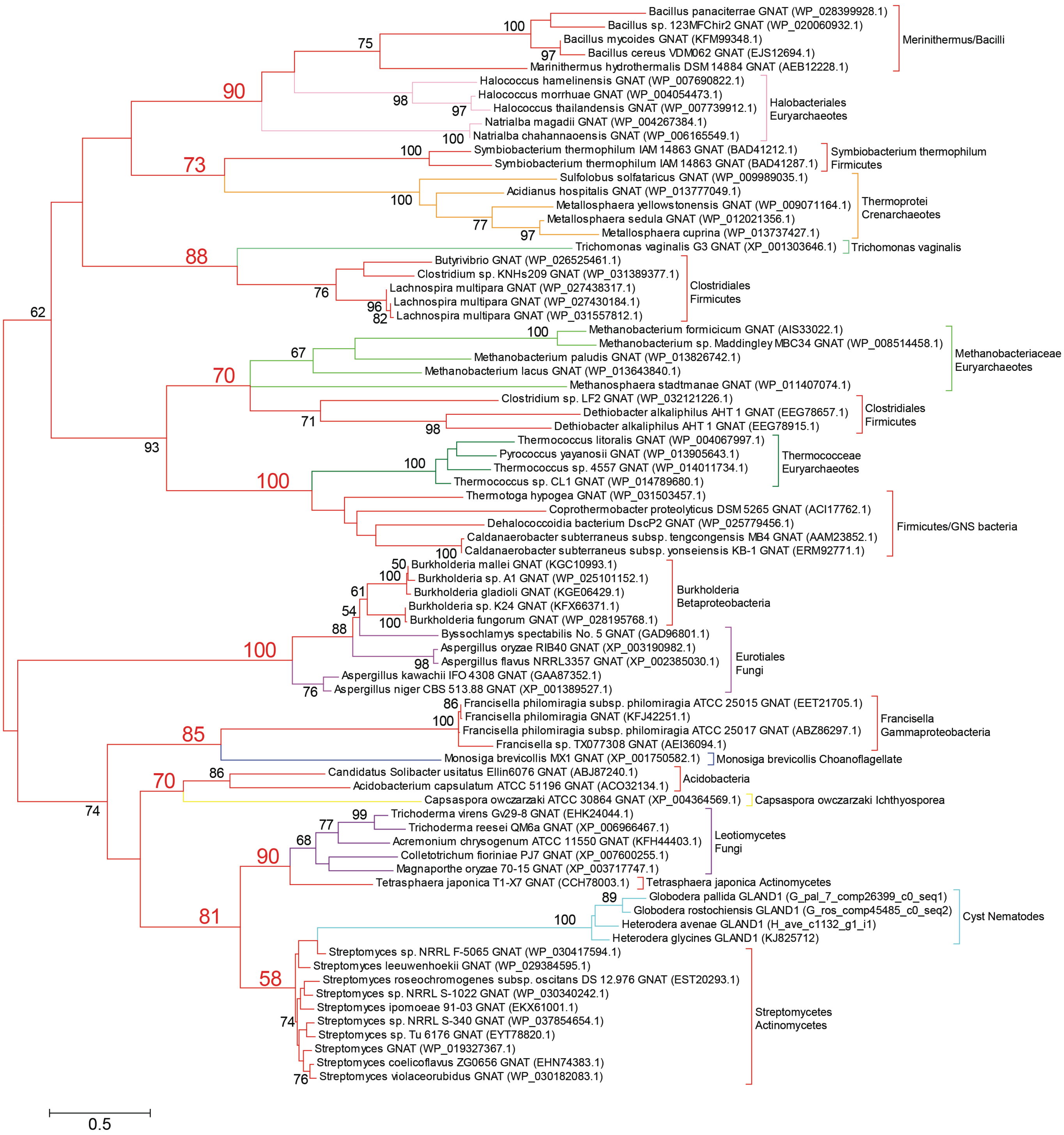
Bacillus panaciterrae GNAT (WP_028399928.1)
Bacillus sp. 123MFChir2 GNAT (WP_020060932.1)
Bacillus mycoides GNAT (KFM99348.1)
Bacillus cereus VDM062 GNAT (EJS12694.1)
Marinithermus hydrothermalis DSM 14884 GNAT (AEB12228.1)
Merinithermus/Bacilli
Halococcus hamelinensis GNAT (WP_007690822.1)
Halococcus morrhuae GNAT (WP_004054473.1)
Halococcus thailandensis GNAT (WP_007739912.1)
Natrialba magadii GNAT (WP_004267384.1)
Natrialba chahannaoensis GNAT (WP_006165549.1)
Halobacteriales Euryarchaeotes
Symbiobacterium thermophilum IAM 14863 GNAT (BAD41212.1)
Symbiobacterium thermophilum IAM 14863 GNAT (BAD41287.1)
Symbiobacterium thermophilum Firmicutes
Sulfolobus solfataricus GNAT (WP_009989035.1)
Acidianus hospitalis GNAT (WP_013777049.1)
Metallosphaera yellowstonensis GNAT (WP_009071164.1)
Metallosphaera sedula GNAT (WP_012021356.1)
Metallosphaera cuprina (WP_013737427.1)
Thermoprotei Crenarchaeotes
Trichomonas vaginalis G3 GNAT (XP_001303646.1)
Trichomonas vaginalis
Butyrivibrio GNAT (WP_026525461.1)
Clostridium sp. KNHs209 GNAT (WP_031389377.1)
Lachnospira multipara GNAT (WP_027438317.1)
Lachnospira multipara GNAT (WP_027430184.1)
Lachnospira multipara GNAT (WP_031557812.1)
Clostridiales Firmicutes
Methanobacterium formicicum GNAT (AIS33022.1)
Methanobacterium sp. Maddingley MBC34 GNAT (WP_008514458.1)
Methanobacterium paludis GNAT (WP_013826742.1)
Methanobacterium lacus GNAT (WP_013643840.1)
Methanosphaera stadtmanae GNAT (WP_011407074.1)
Methanobacteriaceae Euryarchaeotes
Clostridium sp. LF2 GNAT (WP_032121226.1)
Dethiobacter alkaliphilus AHT 1 GNAT (EEG78657.1)
Dethiobacter alkaliphilus AHT 1 GNAT (EEG78915.1)
Clostridiales Firmicutes
Thermococcus litoralis GNAT (WP_004067997.1)
Pyrococcus yayanosii GNAT (WP_013905643.1)
Thermococcus sp. 4557 GNAT (WP_014011734.1)
Thermococcus sp. CL1 GNAT (WP_014789680.1)
Thermococceae Euryarchaeotes
Thermotoga hypogea GNAT (WP_031503457.1)
Coprothermobacter proteolyticus DSM 5265 GNAT (ACI17762.1)
Dehalococcoidia bacterium DscP2 GNAT (WP_025779456.1)
Caldanaerobacter subterraneus subsp. tengcongensis MB4 GNAT (AAM23852.1)
Caldanaerobacter subterraneus subsp. yonseiensis KB-1 GNAT (ERM92771.1)
Firmicutes/GNS bacteria
Burkholderia mallei GNAT (KGC10993.1)
Burkholderia sp. A1 GNAT (WP_025101152.1)
Burkholderia gladioli GNAT (KGE06429.1)
Burkholderia sp. K24 GNAT (KFX66371.1)
Burkholderia fungorum GNAT (WP_028195768.1)
Burkholderia Betaproteobacteria
Byssochlamys spectabilis No. 5 GNAT (GAD96801.1)
Aspergillus oryzae RIB40 GNAT (XP_003190982.1)
Aspergillus flavus NRRL3357 GNAT (XP_002385030.1)
Aspergillus kawachii IFO 4308 GNAT (GAA87352.1)
Aspergillus niger CBS 513.88 GNAT (XP_001389527.1)
Eurotiales Fungi
Francisella philomiragia subsp. philomiragia ATCC 25015 GNAT (EET21705.1)
Francisella philomiragia GNAT (KFJ42251.1)
Francisella philomiragia subsp. philomiragia ATCC 25017 GNAT (ABZ86297.1)
Francisella sp. TX077308 GNAT (AEI36094.1)
Francisella Gammaproteobacteria
Monosiga brevicollis MX1 GNAT (XP_001750582.1)
Monosiga brevicollis Choanoflagellate
Candidatus Solibacter usitatus Ellin6076 GNAT (ABJ87240.1)
Acidobacterium capsulatum ATCC 51196 GNAT (ACO32134.1)
Acidobacteria
Capsaspora owczarzaki ATCC 30864 GNAT (XP_004364569.1)
Capsaspora owczarzaki Ichthyosporea
Trichoderma virens Gv29-8 GNAT (EHK24044.1)
Trichoderma reesei QM6a GNAT (XP_006966467.1)
Acremonium chrysogenum ATCC 11550 GNAT (KFH44403.1)
Colletotrichum fioriniae PJ7 GNAT (XP_007600255.1)
Magnaporthe oryzae 70-15 GNAT (XP_003717747.1)
Leotiomycetes Fungi
Tetrasphaera japonica T1-X7 GNAT (CCH78003.1)
Tetrasphaera japonica Actinomycetes
Globodera pallida GLAND1 (G_pal_7_comp26399_c0_seq1)
Globodera rostochiensis GLAND1 (G_ros_comp45485_c0_seq2)
Heterodera avenae GLAND1 (H_ave_c1132_g1_i1)
Heterodera glycines GLAND1 (KJ825712)
Cyst Nematodes
Streptomyces sp. NRRL F-5065 GNAT (WP_030417594.1)
Streptomyces leeuwenhoekii GNAT (WP_029384595.1)
Streptomyces roseochromogenes subsp. oscitans DS 12.976 GNAT (EST20293.1)
Streptomyces sp. NRRL S-1022 GNAT (WP_030340242.1)
Streptomyces ipomoeae 91-03 GNAT (EKX61001.1)
Streptomyces sp. NRRL S-340 GNAT (WP_037854654.1)
Streptomyces sp. Tu 6176 GNAT (EYT78820.1)
Streptomyces GNAT (WP_019327367.1)
Streptomyces coelicoflavus ZG0656 GNAT (EHN74383.1)
Streptomyces violaceorubidus GNAT (WP_030182083.1)
Streptomycetes Actinomycetes
0.5

Figure S2

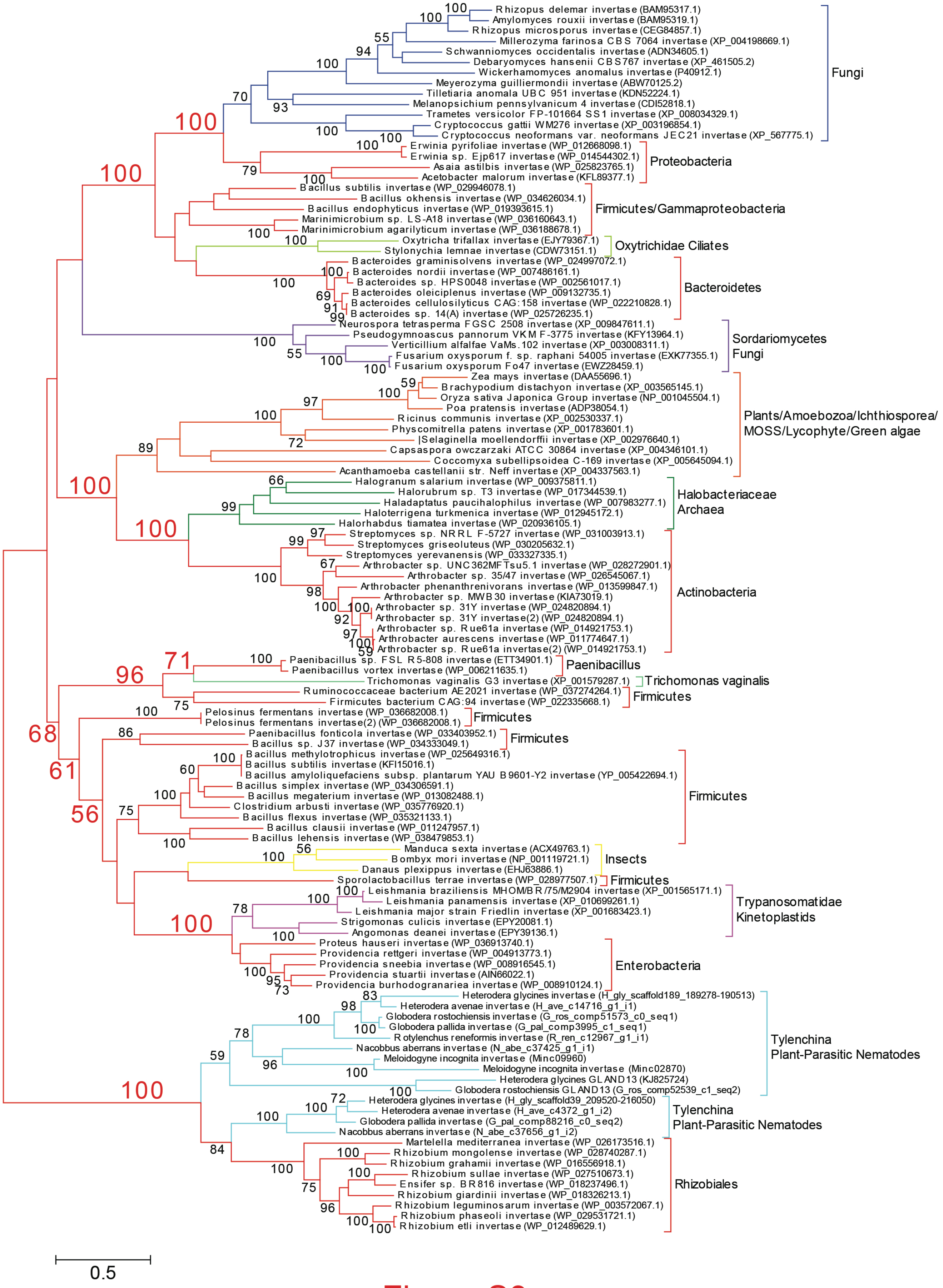

Rhizopus delemar invertase (BAM95317.1)
Amylomyces rouxii invertase (BAM95319.1)
Rhizopus microsporus invertase (CEG84857.1)
Millerozyma farinosa CBS 7064 invertase (XP_004198669.1)
Schwanniomyces occidentalis invertase (ADN34605.1)
Debaryomyces hansenii CBS767 invertase (XP_461505.2)
Wickerhamomyces anomalus invertase (P40912.1)
Meyerozyma guilliermondii invertase (ABW70125.2)
Tilletiaria anomala UBC 951 invertase (KDN52224.1)
Melanopsichium pennsylvanicum 4 invertase (CDI52818.1)
Trametes versicolor FP-101664 SS1 invertase (XP_008034329.1)
Cryptococcus gattii WM276 invertase (XP_003196854.1)
Cryptococcus neoformans var. neoformans JEC21 invertase (XP_567775.1)
Fungi
Erwinia pyrifoliae invertase (WP_012668098.1)
Erwinia sp. Ejp617 invertase (WP_014544302.1)
Asaia astilbis invertase (WP_025823765.1)
Acetobacter malorum invertase (KFL89377.1)
Proteobacteria
Bacillus subtilis invertase (WP_029946078.1)
Bacillus okhensis invertase (WP_034626034.1)
Bacillus endophyticus invertase (WP_019393615.1)
Marinimicrobium sp. LS-A18 invertase (WP_036160643.1)
Marinimicrobium agarilyticum invertase (WP_036188678.1)
Firmicutes/Gammaproteobacteria
Oxytricha trifallax invertase (EJY79367.1)
Stylonychia lemnae invertase (CDW73151.1)
Oxytrichidae Ciliates
Bacteroides graminisolvens invertase (WP_024997072.1)
Bacteroides nordii invertase (WP_007486161.1)
Bacteroides sp. HPS0048 invertase (WP_002561017.1)
Bacteroides oleiciplenus invertase (WP_009132735.1)
Bacteroides cellulosilyticus CAG:158 invertase (WP_022210828.1)
Bacteroides sp. 14(A) invertase (WP_025726235.1)
Bacteroidetes
Neurospora tetrasperma FGSC 2508 invertase (XP_009847611.1)
Pseudogymnoascus pannorum VKM F-3775 invertase (KFY13964.1)
Verticillium alfalfae VaMs.102 invertase (XP_003008311.1)
Fusarium oxysporum f. sp. raphani 54005 invertase (EXK77355.1)
Fusarium oxysporum Fo47 invertase (EWZ28459.1)
Sordariomycetes
Fungi
Zea mays invertase (DAA55696.1)
Brachypodium distachyon invertase (XP_003565145.1)
Oryza sativa Japonica Group invertase (NP_001045504.1)
Poa pratensis invertase (ADP38054.1)
Ricinus communis invertase (XP_002530337.1)
Physcomitrella patens invertase (XP_001783601.1)
|Selaginella moellendorffii invertase (XP_002976640.1)
Capsaspora owczarzaki ATCC 30864 invertase (XP_004346101.1)
Coccomyxa subellipsoidea C-169 invertase (XP_005645094.1)
Acanthamoeba castellanii str. Neff invertase (XP_004337563.1)
Plants/Amoebozoa/Ichthiosporea/
MOSS/Lycophyte/Green algae
Halogranum salarium invertase (WP_009375811.1)
Halorubrum sp. T3 invertase (WP_017344539.1)
Haladaptatus paucihalophilus invertase (WP_007983277.1)
Haloterrigena turkmenica invertase (WP_012945172.1)
Halorhabdus tiamatea invertase (WP_020936105.1)
Halobacteriaceae
Archaea
Streptomyces sp. NRRL F-5727 invertase (WP_031003913.1)
Streptomyces griseoluteus (WP_030205632.1)
Streptomyces yerevanensis (WP_033327335.1)
Arthrobacter sp. UNC362MFTsu5.1 invertase (WP_028272901.1)
Arthrobacter sp. 35/47 invertase (WP_026545067.1)
Arthrobacter phenanthrenivorans invertase (WP_013599847.1)
Arthrobacter sp. MWB30 invertase (KIA73019.1)
Arthrobacter sp. 31Y invertase (WP_024820894.1)
Arthrobacter sp. 31Y invertase(2) (WP_024820894.1)
Arthrobacter sp. Rue61a invertase (WP_014921753.1)
Arthrobacter aurescens invertase (WP_011774647.1)
Arthrobacter sp. Rue61a invertase(2) (WP_014921753.1)
Actinobacteria
Paenibacillus sp. FSL R5-808 invertase (ETT34901.1)
Paenibacillus vortex invertase (WP_006211635.1)
Paenibacillus
Trichomonas vaginalis G3 invertase (XP_001579287.1)
Trichomonas vaginalis
Ruminococcaceae bacterium AE2021 invertase (WP_037274264.1)
Firmicutes bacterium CAG:94 invertase (WP_022335668.1)
Firmicutes
Pelosinus fermentans invertase (WP_036682008.1)
Pelosinus fermentans invertase(2) (WP_036682008.1)
Firmicutes
Paenibacillus fonticola invertase (WP_033403952.1)
Bacillus sp. J37 invertase (WP_034333049.1)
Firmicutes
Bacillus methylotrophicus invertase (WP_025649316.1)
Bacillus subtilis invertase (KFI15016.1)
Bacillus amyloliquefaciens subsp. plantarum YAU B9601-Y2 invertase (YP_005422694.1)
Bacillus simplex invertase (WP_034306591.1)
Bacillus megaterium invertase (WP_013082488.1)
Clostridium arbusti invertase (WP_035776920.1)
Bacillus flexus invertase (WP_035321133.1)
Bacillus clausii invertase (WP_011247957.1)
Bacillus lehensis invertase (WP_038479853.1)
Firmicutes
Manduca sexta invertase (ACX49763.1)
Bombyx mori invertase (NP_001119721.1)
Danaus plexippus invertase (EHJ63886.1)
Insects
Sporolactobacillus terrae invertase (WP_028977507.1)
Firmicutes
Leishmania braziliensis MHOM/BR/75/M2904 invertase (XP_001565171.1)
Leishmania panamensis invertase (XP_010699261.1)
Leishmania major strain Friedlin invertase (XP_001683423.1)
Strigomonas culicis invertase (EPY20081.1)
Angomonas deanei invertase (EPY39136.1)
Trypanosomatidae
Kinetoplastids
Proteus hauseri invertase (WP_036913740.1)
Providencia rettgeri invertase (WP_004913773.1)
Providencia sneebia invertase (WP_008916545.1)
Providencia stuartii invertase (AIN66022.1)
Providencia burhodogranariea invertase (WP_008910124.1)
Enterobacteria
Heterodera glycines invertase (H_gly_scaffold189_189278-190513)
Heterodera avenae invertase (H_ave_c14716_g1_i1)
Globodera rostochiensis invertase (G_ros_comp51573_c0_seq1)
Globodera pallida invertase (G_pal_comp3995_c1_seq1)
Rotylenchus reneformis invertase (R_ren_c12967_g1_i1)
Nacobbus aberrans invertase (N_abe_c37425_g1_i1)
Meloidogyne incognita invertase (Minc09960)
Meloidogyne incognita invertase (Minc02870)
Heterodera glycines GLAND13 (KJ825724)
Globodera rostochiensis GLAND13 (G_ros_comp52539_c1_seq2)
Tylenchina
Plant-Parasitic Nematodes
Heterodera glycines invertase (H_gly_scaffold39_209520-216050)
Heterodera avenae invertase (H_ave_c4372_g1_i2)
Globodera pallida invertase (G_pal_comp88216_c0_seq2)
Nacobbus aberrans invertase (N_abe_c37656_g1_i2)
Tylenchina
Plant-Parasitic Nematodes
Martelella mediterranea invertase (WP_026173516.1)
Rhizobium mongolense invertase (WP_028740287.1)
Rhizobium grahamii invertase (WP_016556918.1)
Rhizobium sullae invertase (WP_027510673.1)
Ensifer sp. BR816 invertase (WP_018237496.1)
Rhizobium giardinii invertase (WP_018326213.1)
Rhizobium leguminosarum invertase (WP_003572067.1)
Rhizobium phaseoli invertase (WP_029531721.1)
Rhizobium etli invertase (WP_012489629.1)
Rhizobiales
0.5

Figure S3

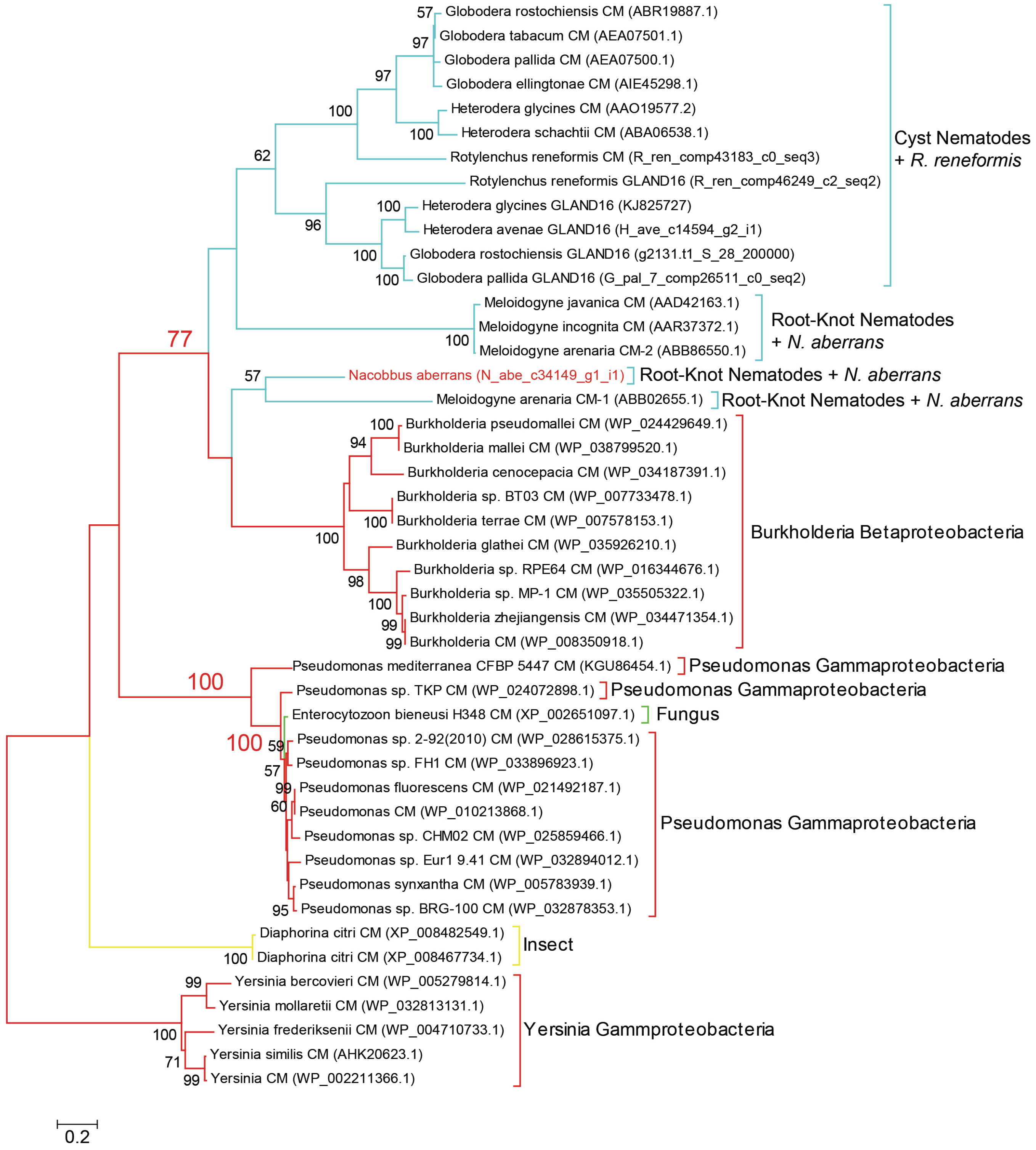

Globodera rostochiensis CM (ABR19887.1)
Globodera tabacum CM (AEA07501.1)
Globodera pallida CM (AEA07500.1)
Globodera ellingtonae CM (AIE45298.1)
Heterodera glycines CM (AAO19577.2)
Heterodera schachtii CM (ABA06538.1)
Rotylenchus reneformis CM (R_ren_comp43183_c0_seq3)
Rotylenchus reneformis GLAND16 (R_ren_comp46249_c2_seq2)
Heterodera glycines GLAND16 (KJ825727)
Heterodera avenae GLAND16 (H_ave_c14594_g2_i1)
Globodera rostochiensis GLAND16 (g2131.t1_S_28_200000)
Globodera pallida GLAND16 (G_pal_7_comp26511_c0_seq2)
Cyst Nematodes + *R. reneformis*
Meloidogyne javanica CM (AAD42163.1)
Meloidogyne incognita CM (AAR37372.1)
Meloidogyne arenaria CM-2 (ABB86550.1)
Root-Knot Nematodes + *N. aberrans*
Nacobbus aberrans (N_abe_c34149_g1_i1)
Root-Knot Nematodes + *N. aberrans*
Meloidogyne arenaria CM-1 (ABB02655.1)
Root-Knot Nematodes + *N. aberrans*
Burkholderia pseudomallei CM (WP_024429649.1)
Burkholderia mallei CM (WP_038799520.1)
Burkholderia cenocepacia CM (WP_034187391.1)
Burkholderia sp. BT03 CM (WP_007733478.1)
Burkholderia terrae CM (WP_007578153.1)
Burkholderia glathei CM (WP_035926210.1)
Burkholderia sp. RPE64 CM (WP_016344676.1)
Burkholderia sp. MP-1 CM (WP_035505322.1)
Burkholderia zhejiangensis CM (WP_034471354.1)
Burkholderia CM (WP_008350918.1)
Burkholderia Betaproteobacteria
Pseudomonas mediterranea CFBP 5447 CM (KGU86454.1)
Pseudomonas Gammaproteobacteria
Pseudomonas sp. TKP CM (WP_024072898.1)
Pseudomonas Gammaproteobacteria
Enterocytozoon bieneusi H348 CM (XP_002651097.1)
Fungus
Pseudomonas sp. 2-92(2010) CM (WP_028615375.1)
Pseudomonas sp. FH1 CM (WP_033896923.1)
Pseudomonas fluorescens CM (WP_021492187.1)
Pseudomonas CM (WP_010213868.1)
Pseudomonas sp. CHM02 CM (WP_025859466.1)
Pseudomonas sp. Eur1 9.41 CM (WP_032894012.1)
Pseudomonas synxantha CM (WP_005783939.1)
Pseudomonas sp. BRG-100 CM (WP_032878353.1)
Pseudomonas Gammaproteobacteria
Diaphorina citri CM (XP_008482549.1)
Diaphorina citri CM (XP_008467734.1)
Insect
Yersinia bercovieri CM (WP_005279814.1)
Yersinia mollaretii CM (WP_032813131.1)
Yersinia frederiksenii CM (WP_004710733.1)
Yersinia similis CM (AHK20623.1)
Yersinia CM (WP_002211366.1)
Yersinia Gammproteobacteria
0.2

Figure S4

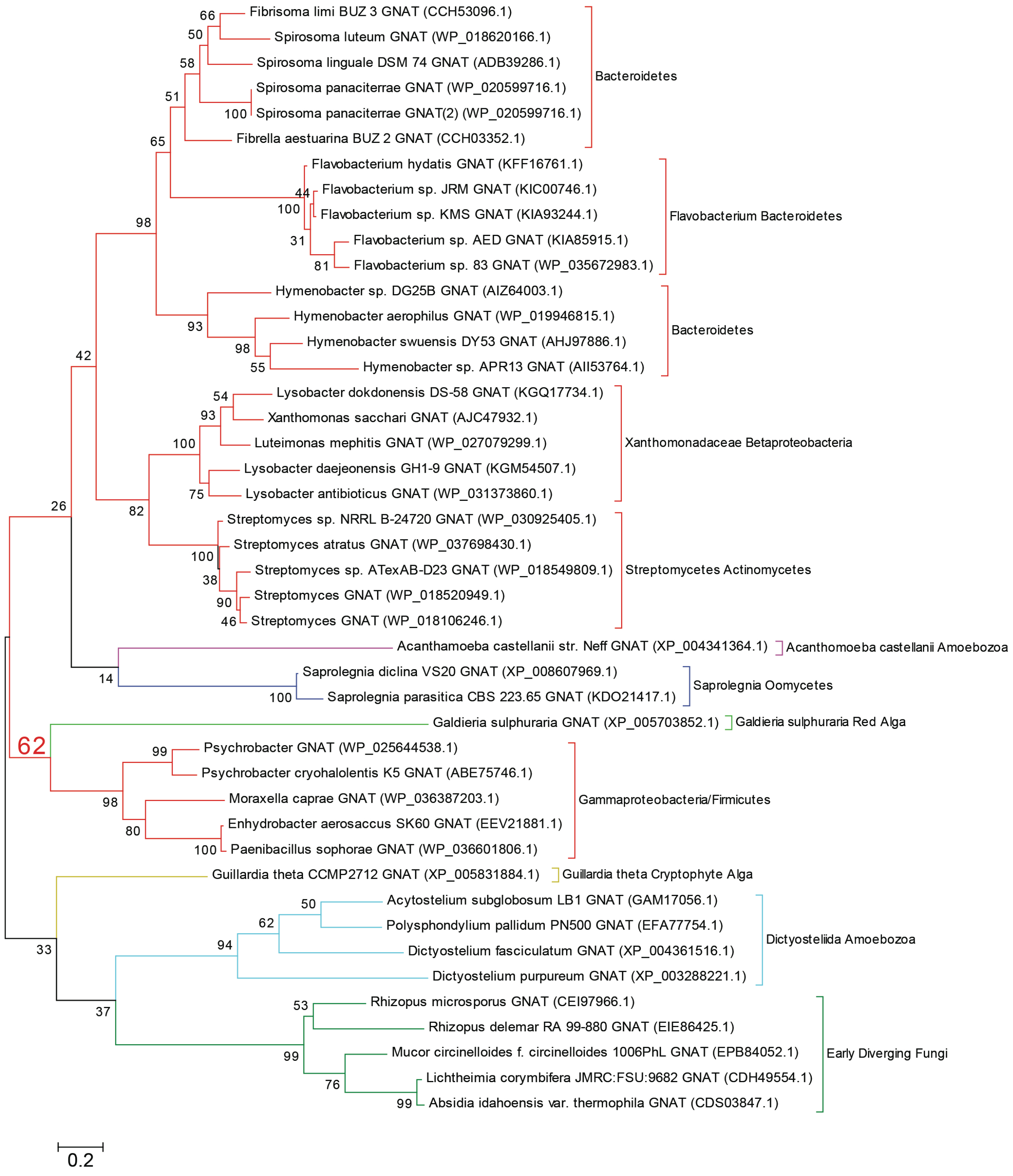
Fibrisoma limi BUZ 3 GNAT (CCH53096.1)
Spirosoma luteum GNAT (WP_018620166.1)
Spirosoma linguale DSM 74 GNAT (ADB39286.1)
Spirosoma panaciterrae GNAT (WP_020599716.1)
Spirosoma panaciterrae GNAT(2) (WP_020599716.1)
Fibrella aestuarina BUZ 2 GNAT (CCH03352.1)
Bacteroidetes
Flavobacterium hydatis GNAT (KFF16761.1)
Flavobacterium sp. JRM GNAT (KIC00746.1)
Flavobacterium sp. KMS GNAT (KIA93244.1)
Flavobacterium sp. AED GNAT (KIA85915.1)
Flavobacterium sp. 83 GNAT (WP_035672983.1)
Flavobacterium Bacteroidetes
Hymenobacter sp. DG25B GNAT (AIZ64003.1)
Hymenobacter aerophilus GNAT (WP_019946815.1)
Hymenobacter swuensis DY53 GNAT (AHJ97886.1)
Hymenobacter sp. APR13 GNAT (AII53764.1)
Bacteroidetes
Lysobacter dokdonensis DS-58 GNAT (KGQ17734.1)
Xanthomonas sacchari GNAT (AJC47932.1)
Luteimonas mephitis GNAT (WP_027079299.1)
Lysobacter daejeonensis GH1-9 GNAT (KGM54507.1)
Lysobacter antibioticus GNAT (WP_031373860.1)
Xanthomonadaceae Betaproteobacteria
Streptomyces sp. NRRL B-24720 GNAT (WP_030925405.1)
Streptomyces atratus GNAT (WP_037698430.1)
Streptomyces sp. ATexAB-D23 GNAT (WP_018549809.1)
Streptomyces GNAT (WP_018520949.1)
Streptomyces GNAT (WP_018106246.1)
Streptomycetes Actinomycetes
Acanthamoeba castellanii str. Neff GNAT (XP_004341364.1)
Acanthomoeba castellanii Amoebozoa
Saprolegnia diclina VS20 GNAT (XP_008607969.1)
Saprolegnia parasitica CBS 223.65 GNAT (KDO21417.1)
Saprolegnia Oomycetes
Galdieria sulphuraria GNAT (XP_005703852.1)
Galdieria sulphuraria Red Alga
Psychrobacter GNAT (WP_025644538.1)
Psychrobacter cryohalolentis K5 GNAT (ABE75746.1)
Moraxella caprae GNAT (WP_036387203.1)
Enhydrobacter aerosaccus SK60 GNAT (EEV21881.1)
Paenibacillus sophorae GNAT (WP_036601806.1)
Gammaproteobacteria/Firmicutes
Guillardia theta CCMP2712 GNAT (XP_005831884.1)
Guillardia theta Cryptophyte Alga
Acytostelium subglobosum LB1 GNAT (GAM17056.1)
Polysphondylium pallidum PN500 GNAT (EFA77754.1)
Dictyostelium fasciculatum GNAT (XP_004361516.1)
Dictyostelium purpureum GNAT (XP_003288221.1)
Dictyosteliida Amoebozoa
Rhizopus microsporus GNAT (CEI97966.1)
Rhizopus delemar RA 99-880 GNAT (EIE86425.1)
Mucor circinelloides f. circinelloides 1006PhL GNAT (EPB84052.1)
Lichtheimia corymbifera JMRC:FSU:9682 GNAT (CDH49554.1)
Absidia idahoensis var. thermophila GNAT (CDS03847.1)
Early Diverging Fungi
66
50
58
51
100
65
44
100
31
81
98
93
98
55
42
54
93
100
75
26
82
100
38
90
46
14
100
62
99
98
80
100
50
62
94
33
37
53
99
76
99
0.2

Figure S5

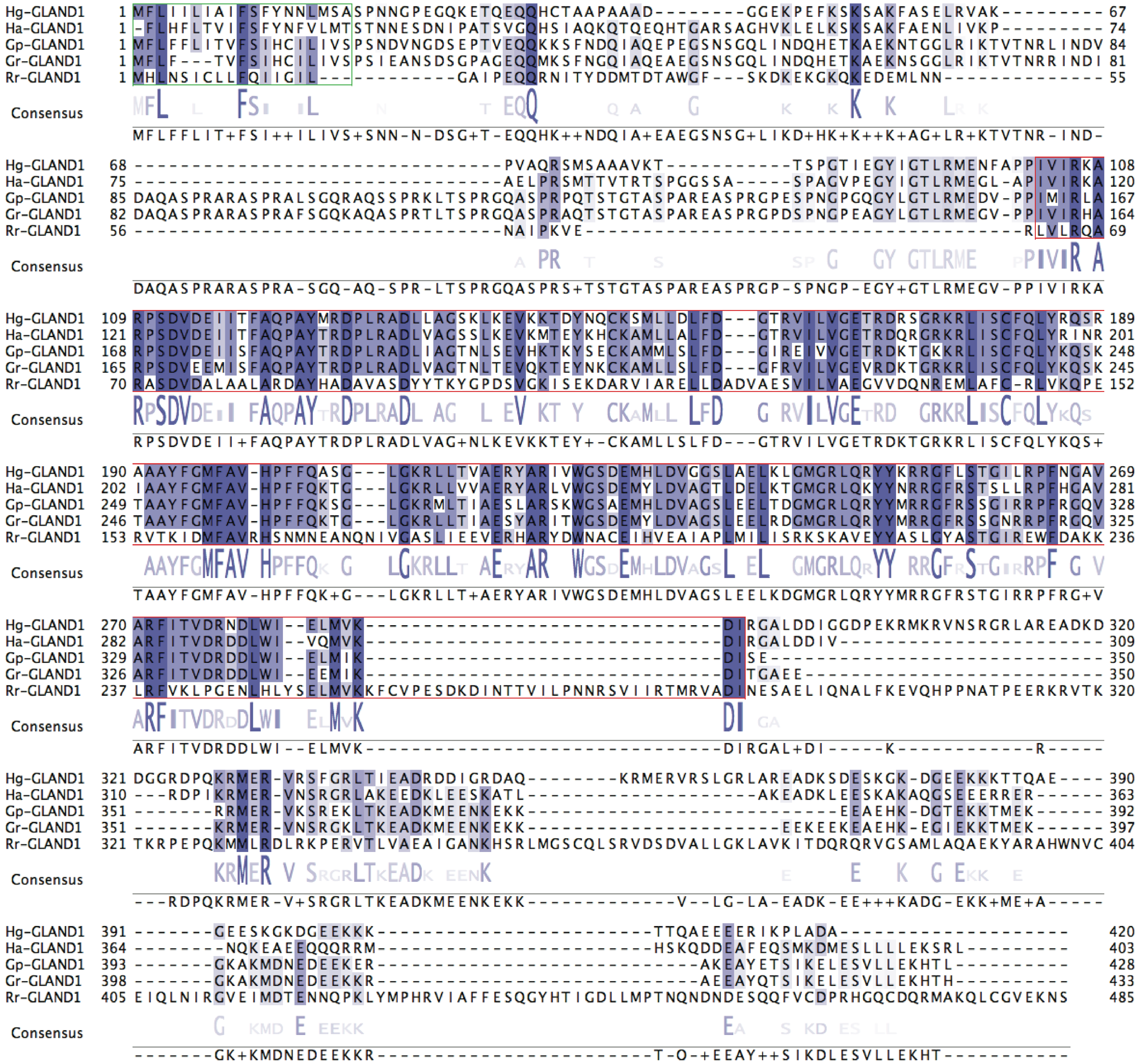
Hg-GLAND1 1 MFLIILAIFSFYNNLMSASPNNGPEGQKETQEQQHCTAAPAAAD--------GGEKPEFKSKSAKFASELRVAK--------- 67
Ha-GLAND1 1 -FLHFLTVIFSFYNFVLMTSTNNESDNIPATSVGQHSIAQKQTQEQHTGARSAGHVKLELKSKSAKFAENLIVKP--------- 74
Gp-GLAND1 1 MFLFFLITVFSIHCILIVSPSNDVNGDSEPTVEQQKKSFNDQIAQEPEGSNSGQLINDQHETKAEKNTGGLRIKTVTNRLINDV 84
Gr-GLAND1 1 MFLF---TVFSIHCILIVSPSIEANSDSGPAGEQQMKSFNGQIAQEAEGSNSGQLINDQHETKAEKNSGGLRIKTVTNRRINDI 81
Rr-GLAND1 1 MHLNSICLLFQIIGIL---------GAIPEQQRNITYDDMTDTAWGF---SKDKEKGKQKEDEMLNN-------------- 55
Consensus
MFLFFLIT+FSI++ILIVS+SNN-N-DSG+T-EQQHK++NDQIA+EAEGSNSG+LIKD+HK+K++K+AG+LR+KTVTNR-IND-
Hg-GLAND1 68 ---------------------------------PVAQRSMSAAAVKT-----------TSPGTIEGYIGTLRMENFAPPIVIRKA 108
Ha-GLAND1 75 --------------------------------AELPRSMTTVTRTSPGGSSA-----SPAGVPEGYIGTLRMEGL-APIVIRKA 120
Gp-GLAND1 85 DAQASPRARASPRALSGQRAQSSPRKLTSPRGQASPRPQTSTGTASPAREASPRGPESPNGPGQGYLGTLRMEDV-PPIMIRLA 167
Gr-GLAND1 82 DAQASPRARASPRAFSGQKAQASPRTLTSPRGQASPRAQTSTGTASPAREASPRGPDSPNGPEAGYLGTLRMEGV-PPIVIRHA 164
Rr-GLAND1 56 --------------------------------NAIPKVE-------------------------------------RLVLRQA 69
Consensus
DAQASPRARASPRA-SGQ-AQ-SPR-LTSPRGQASPRS+TSTGTASPAREASPRGP-SPNGP-EGY+GTLRMEGV-PPIVIRKA
Hg-GLAND1 109 RPSDVDEIITFAQPAYMRDPLRADLLAGSKLKEVKKTDYNQCKSMLLDLFD---GTRVILVGETRDRSGRKRLISCFQLYRQSR 189
Ha-GLAND1 121 RPSDVDEIITFAQPAYTRDPLRADLVAGSSLKEVKMTEYKHCKAMLLALFD---GTRVILVGETRDQRGRKRLISCFQLYRINR 201
Gp-GLAND1 168 RPSDVDEIISFAQPAYTRDPLRADLIAGTNLSEVHKTKYSECKAMMLSLFD---GIREIVVGETRDKTGKKRLISCFQLYKQSK 248
Gr-GLAND1 165 RPSDVEEMISFAQPAYTRDPLRADLVAGTNLTEVQKTEYNKCKAMLLSLFD---GFRVILVGEVRDKTGRKRLISCFQLYKQSK 245
Rr-GLAND1 70 RASDVDALAALARDAYHADAVASDYYTKYGPDSVGKISEKDARVIARELLDADVAESVILVAEGVVDQNREMLAFC-RLVKQPE 152
Consensus
RPSDVDEII+FAQPAYTRDPLRADLVAG+NLKEVKKTEY+-CKAMLLSLFD---GTRVILVGETRDKTGRKRLISCFQLYKQS+
Hg-GLAND1 190 AAAYFGMFAV-HPFFQASG---LGKRLLTVAERYARIVWGSDEMHLDVGGSLAELKLGMGRLQRYYKRRGFLSTGILRPFNGAV 269
Ha-GLAND1 202 IAAYFGMFAV-HPFFQKTG---LGKRLLVVAERYARLVWGSDEMYLDVACTLDELKTGMGRLQKYYNRRGFRSTSLLRPFHGAV 281
Gp-GLAND1 249 TAAYFGMFAV-HPFFQKSG---LGKRMLTIAESLARSKWGSAEMHLDVAGSLEELTDGMGRLQRYYMRRGFRSSGIRRPFRGQV 328
Gr-GLAND1 246 TAAYFGMFAV-HPFFQKTG---LGKRLLTIAESYARITWGSDEMYLDVAGSLEELRDGMGRLQRYYMRRGFRSSGNRRPFRGQV 325
Rr-GLAND1 153 RVTKIDMFAVRHSNMNEANQNIVGASLIEEVERHARYDWNACEIHVEAIAPLMILISRKSKAVEYYASLGYASTGIREWFDAKK 236
Consensus
TAAYFGMFAV-HPFFQK+G---LGKRLLT+AERYARIVWGSDEMHLDVAGSLEELKDGMGRLQRYYMRRGFRSTGIRRPFRG+V
Hg-GLAND1 270 ARFITVDRNDLWI--ELMVK------------------------------DIRGALDDIGGDPEKRMKRVNSRGRLAREADKD 320
Ha-GLAND1 282 ARFITVDRDDLWI--VQMVK------------------------------DIRGALDDIV----------------------- 309
Gp-GLAND1 329 ARFITVDRDDLWI--ELMIK------------------------------DISE----------------------------- 350
Gr-GLAND1 326 ARFITVDRDDLWI--EEMIK------------------------------DITGAEE-------------------------- 350
Rr-GLAND1 237 LRFVKLPGENLHLYSELMVKKFCVPESDKDINTTVILPNNRSVIIRTMRVADINESAELIQNALFKEVQHPPNATPEERKRVTK 320
Consensus
ARFITVDRDDLWI--ELMVK------------------------------DIRGAL+DI-----K------------R-----
Hg-GLAND1 321 DGGRDPQKRMER-VRSFGRLTIEADRDDIGRDAQ--------KRMERVRSLGRLAREADKSDESKGK-DGEEKKKTTQAE---- 390
Ha-GLAND1 310 ---RDPIKRMER-VNSRGRLAKEEDKLEESKATL---------------------AKEADKLEESKAKAQGSEEERRER------ 363
Gp-GLAND1 351 -------RRMER-VKSREKLTKEADKMEENKEKK---------------------------EEAEHK-DGTEKKTMEK------ 392
Gr-GLAND1 351 -------KRMER-VNSRGKLTKEADKMEENKEKK----------------------EEKEEKEAEHK-EGIEKKTMEK------ 397
Rr-GLAND1 321 TKRPEPQKMMLRDLRKPERVTLVAEAIGANKHSRLMGSCQLSRVDSDVALLGKLAVKITDQRQRVGSAMLAQAEKYARAHWNVC 404
Consensus
---RDPQKRMER-V+SRGRLTKEADKMEENKEKK-------------V--LG-LA-EADK-EE+++KADG-EKK+ME+A-----
Hg-GLAND1 391 -------GEESKGKDGEEKKK-----------------------TTQAEEEERIKPLADA------------------ 420
Ha-GLAND1 364 --------NQKEAEEQQQRRM-----------------------HSKQDDEAFEQSMKDMESLLLLEKSRL--------- 403
Gp-GLAND1 393 -------GKAKMDNEDEEKER--------------------------AKEAYETSIKELESVLLEKHTL---------- 428
Gr-GLAND1 398 -------GKAKMDNEDEEKKR--------------------------AEEAYQTSIKELESVLLEKHTH---------- 433
Rr-GLAND1 405 EIQLNIRGVEIMDTENNQPKLYMPHRVIAFFESQGYHTIGDLLMPTNQNDNDESQQFVCDPRHGQCDQRMAKQLCGVEKNS 485
Consensus
-------GK+KMDNEDEEKKR-----------------------T-O-+EEAY++SIKDLESVLLEKHT-----------

Figure S6

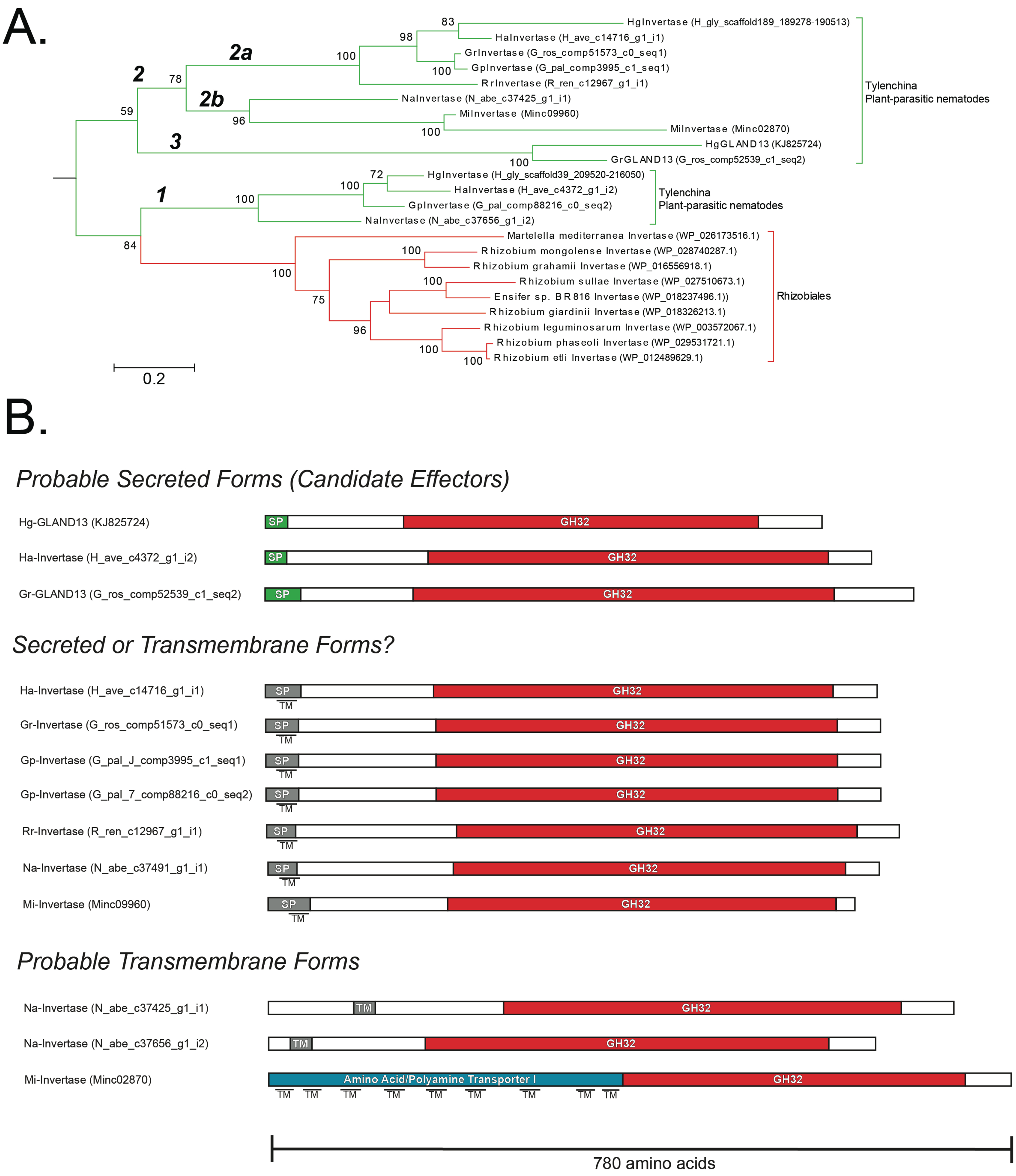
A.
83
98
100
100
78
59
96
100
100
2a
2
2b
3
HgInvertase (H_gly_scaffold189_189278-190513)
HaInvertase (H_ave_c14716_g1_i1)
GrInvertase (G_ros_comp51573_c0_seq1)
GpInvertase (G_pal_comp3995_c1_seq1)
RrInvertase (R_ren_c12967_g1_i1)
NaInvertase (N_abe_c37425_g1_i1)
MiInvertase (Minc09960)
MiInvertase (Minc02870)
HgGLAND13 (KJ825724)
GrGLAND13 (G_ros_comp52539_c1_seq2)
Tylenchina
Plant-parasitic nematodes
72
100
100
1
84
HgInvertase (H_gly_scaffold39_209520-216050)
HaInvertase (H_ave_c4372_g1_i2)
GpInvertase (G_pal_comp88216_c0_seq2)
NaInvertase (N_abe_c37656_g1_i2)
Tylenchina
Plant-parasitic nematodes
100
100
75
100
96
100
100
Martelella mediterranea Invertase (WP_026173516.1)
Rhizobium mongolense Invertase (WP_028740287.1)
Rhizobium grahamii Invertase (WP_016556918.1)
Rhizobium sullae Invertase (WP_027510673.1)
Ensifer sp. BR816 Invertase (WP_018237496.1))
Rhizobium giardinii Invertase (WP_018326213.1)
Rhizobium leguminosarum Invertase (WP_003572067.1)
Rhizobium phaseoli Invertase (WP_029531721.1)
Rhizobium etli Invertase (WP_012489629.1)
Rhizobiales
0.2
B.
Probable Secreted Forms (Candidate Effectors)
Hg-GLAND13 (KJ825724)
Ha-Invertase (H_ave_c4372_g1_i2)
Gr-GLAND13 (G_ros_comp52539_c1_seq2)
SP
GH32
Secreted or Transmembrane Forms?
Ha-Invertase (H_ave_c14716_g1_i1)
Gr-Invertase (G_ros_comp51573_c0_seq1)
Gp-Invertase (G_pal_J_comp3995_c1_seq1)
Gp-Invertase (G_pal_7_comp88216_c0_seq2)
Rr-Invertase (R_ren_c12967_g1_i1)
Na-Invertase (N_abe_c37491_g1_i1)
Mi-Invertase (Minc09960)
SP
TM
GH32
Probable Transmembrane Forms
Na-Invertase (N_abe_c37425_g1_i1)
Na-Invertase (N_abe_c37656_g1_i2)
Mi-Invertase (Minc02870)
TM
GH32
Amino Acid/Polyamine Transporter I
780 amino acids

Figure S7

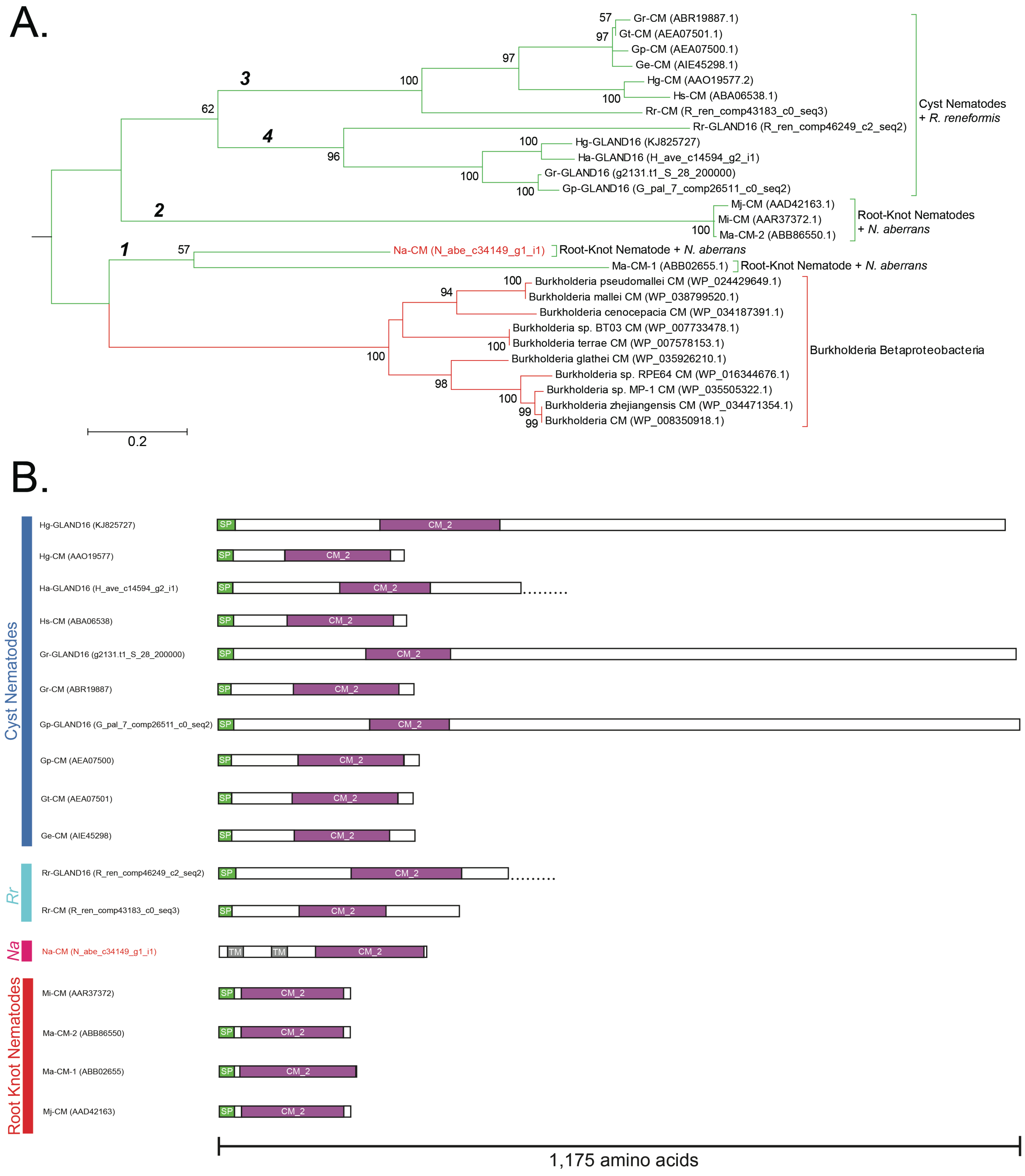
A.
Gr-CM (ABR19887.1)
Gt-CM (AEA07501.1)
Gp-CM (AEA07500.1)
Ge-CM (AIE45298.1)
Hg-CM (AAO19577.2)
Hs-CM (ABA06538.1)
Rr-CM (R_ren_comp43183_c0_seq3)
Rr-GLAND16 (R_ren_comp46249_c2_seq2)
Hg-GLAND16 (KJ825727)
Ha-GLAND16 (H_ave_c14594_g2_i1)
Gr-GLAND16 (g2131.t1_S_28_200000)
Gp-GLAND16 (G_pal_7_comp26511_c0_seq2)
Cyst Nematodes + R. reneformis
Mj-CM (AAD42163.1)
Mi-CM (AAR37372.1)
Ma-CM-2 (ABB86550.1)
Root-Knot Nematodes + N. aberrans
Na-CM (N_abe_c34149_g1_i1)
Root-Knot Nematode + N. aberrans
Ma-CM-1 (ABB02655.1)
Root-Knot Nematode + N. aberrans
Burkholderia pseudomallei CM (WP_024429649.1)
Burkholderia mallei CM (WP_038799520.1)
Burkholderia cenocepacia CM (WP_034187391.1)
Burkholderia sp. BT03 CM (WP_007733478.1)
Burkholderia terrae CM (WP_007578153.1)
Burkholderia glathei CM (WP_035926210.1)
Burkholderia sp. RPE64 CM (WP_016344676.1)
Burkholderia sp. MP-1 CM (WP_035505322.1)
Burkholderia zhejiangensis CM (WP_034471354.1)
Burkholderia CM (WP_008350918.1)
Burkholderia Betaproteobacteria
0.2
B.
Cyst Nematodes
Hg-GLAND16 (KJ825727)
Hg-CM (AAO19577)
Ha-GLAND16 (H_ave_c14594_g2_i1)
Hs-CM (ABA06538)
Gr-GLAND16 (g2131.t1_S_28_200000)
Gr-CM (ABR19887)
Gp-GLAND16 (G_pal_7_comp26511_c0_seq2)
Gp-CM (AEA07500)
Gt-CM (AEA07501)
Ge-CM (AIE45298)
Rr
Rr-GLAND16 (R_ren_comp46249_c2_seq2)
Rr-CM (R_ren_comp43183_c0_seq3)
Na
Na-CM (N_abe_c34149_g1_i1)
Root Knot Nematodes
Mi-CM (AAR37372)
Ma-CM-2 (ABB86550)
Ma-CM-1 (ABB02655)
Mj-CM (AAD42163)
SP
CM_2
TM
1,175 amino acids

Figure S8
