# Supplementary material for: Horizontal gene transfer of acetyltransferases, invertases and chorismate mutases from different bacteria to diverse recipients
Source: BMC Evol Biol. 2016 Apr 12;16:74. doi: 10.1186/s12862-016-0651-y (PMC4828791; doi:10.1186/s12862-016-0651-y)
Supplement: Additional file 5: — Complete model selection test results for phylogenetic analysis of the CMs. (PDF 123 kb) [file 12862_2016_651_MOESM5_ESM.pdf]

Horizontal gene transfer of *acetyltransferases, invertases* and *chorismate mutases* from different bacteria to diverse recipients

Jason B. Noon[1] and Thomas J. Baum[1]*

[1]Department of Plant Pathology and Microbiology, Iowa State University Ames, IA 50011, U.S.A.

Email addresses: JBN, jnoon15@iastate.edu; TJB, tbaum@iastate.edu

*Corresponding author

Supplementary Text

Evolution of *GNATs*, *INVs* and *CMs* in Hoplolaimina following HGT from rhizosphere bacteria

The family of *GLAND1*s (*FAM7 GNATs*) appeared to be rather simple in that, as mentioned in the main text, it seemed to have originated via horizontal gene transfer (HGT) in Hoplolaimina clade B (fig. 2A). We only identified a single homolog in each Hoplolaimina plant-parasitic nematode (PPN; cyst nematodes and reniform nematode *Rotylenchulus reniformis*) (see fig. S2), and with their highly significant sequence similarities (fig. S6), this strongly suggested orthology. Also, the protein sequences were found to be highly similar within and near their GNAT domains, and signal peptides were identified at the N-termini of all orthologs (fig. S6), suggesting that all may be effectors in the respective Hoplolaimina PPN. On the other hand, sequences N- and C-terminal to the GNAT domain in all orthologs are highly variable, suggesting that these regions of the protein are evolving relatively fast. Given that *Hg-GLAND1* was found to encode a candidate effector [7], the latter finding lead us to speculate that those regions might be involved in binding to target proteins, possibly for acetylation.

We next inspected the *invertase* (*INV*) gene family. In the raw phylogenetic tree shown in figure S3, from which we constructed a subtree shown in figure S7, there appeared to be a complexity of gene duplications and subsequent losses (however we consider that gene loss may simply be an artifact of incomplete genomic or transcriptomic sequences, or lack of detected expression from the evaluated transcriptomes). For instance, three different, highly supported clusters were found (fig. S7). Cluster 1 is in a nested, highly supported monophyletic group with the donor bacteria Rhizobiales and consists of orthologs—indicated by bootstrap values > 95—from *Heterodera glycines*, *Heterodera avenae*, *Globodera pallida* and *Nacobbus aberrans*, strongly suggesting that this cluster contains the original transferred genes (i.e., xenologs). Interestingly, *G. pallida* and *N. aberrans* INVs in cluster 1 are the homologs that resulted in %GC contents significantly greater than expected and within the range of Rhizobiales (fig. 7B), further supporting xenology of cluster 1 with Rhizobiales INVs.

Clusters 2 and 3 are in a monophyletic group, and although not highly supported (bootstrap of only 59), suggest two duplications (fig. S7). The first duplication resulted in clusters 1 and 2/3 and probably occurred prior to divergence of Hoplolaimina clade 2 due to the presence of both Hoplolaimina clade A and B PPN in these clusters (see fig. 1B). The second duplication probably occurred in the cyst nematode lineage since only *H. glycines* and *G. rostochiensis* are found in cluster 3, which contains the *GLAND13s*. Also, we identified another duplication within *M. incognita* (cluster 2b; fig. S7), which probably occurred within the root-knot nematode lineage and before its divergence due to the relatively long branch lengths (sequence divergence)—additional root-knot nematode sequences could be analyzed for that duplication in future studies. These interpretations would likewise propose the following gene losses: First, the cluster 1 orthologs, and thus the likely xenologs of the rhizobacterial *INVs*, may have been lost in root-knot nematodes (at least in *M. incognita*), *R. reniformis* and *G. rostochiensis*. Second, the cluster 3 *GLAND13* may have been lost from *G. pallida* and *H. avenae*, and possibly other *Globodera* and *Heterodera* species, with no apparent losses from cluster 2 (fig. S7). As mentioned above, it is still possible that some of these alleged gene losses after the duplication events are actually due to insufficient sequence data.

As shown in figure S7, all INV homologs were found to consist of predominantly a single GH32 domain (InterPro: IPR001362). On the other hand, we were surprised to find extensive variation from signal peptide predictions (see Methods). The following three classes were found: First, we found probable secreted forms containing predicted N-terminal signal peptides with no overlap with transmembrane (TM) regions, which contained both GLAND13s, consistent with their candidacy as effectors, at least in *Heterodera*, as well as the Ha-INV from cluster 1 (fig. S7). Second, homologs were found to be uncertain whether secreted or TM forms due to overlapping predictions of signal peptides and TM regions, which contained over half of the GH32 homologs (fig. S7). Third, and of particular interest, we found probable TM forms containing predicted, non-N-terminal TM regions without predicted signal peptides. Firstly, class 2 is not an uncommon challenge for signal peptide predictions as both signal peptides and TM regions contain similar stretches of hydrophobic amino acids; referred to as the H-region in signal peptides [50]. We found that these class 2 INVs are all positive for predicted signal peptides only when the assumption is provided that TM regions are absent, but not when TM regions are considered, and all are predicted positive for TM regions regardless of criteria, further adding to the uncertainty. However, the TM regions predicted in all class 2 homologs overlap almost perfectly where the signal peptide H-region is typically found (fig. S7). Thus, we believed that it was more likely that these are degenerate signal peptides, and therefore also possible secreted forms, rather than TM regions, but this of course will have to be empirically tested in future studies to be certain.

The particular interest in the class 3 INVs (fig. S7) is threefold: Firstly, these probable TM forms are only found in false root-knot nematodes and root-knot nematodes, and thus appear to have formed in Hoplolaimina clade A (see fig. 1B). Secondly, the class 3 Mi-INV contains a GH32 domain with an amino acid/polyamine transporter 1 (aka AA permease) domain (InterPro: IPR002293) appended at the N-terminus. The AA permease domain comprises almost the entire N-terminal half of the protein and contains at least

nine TM regions. Also, in a separate blastp search with the Mi-INV AA permease domain as query, we identified highly significant matches to numerous AA permeases from nematodes, of which all were found to consist entirely of single AA permease domains (data not shown). Noteworthy, this class 3 Mi-INV (Minc02870) is the highly diverged *M. incognita* paralog in cluster 2b (fig. S7). Taken together, this suggested that in the root-knot nematode lineage, the GH32 domain acquired from rhizobacteria was appended to an AA permease domain from the nematode genome. Thirdly, although the GH32 domains from the class 3 *N. aberrans* homologs are predicted as non-cytoplasmic suggesting extracellular processing of sucrose within the nematode, the GH32 domain from the *M. incognita* homolog is predicted to be on the cytoplasmic surface (data not shown). Accordingly, we speculate that the class 3 Mi-INV might use a cotransport mechanism (either symport or antiport) characteristic of AA permeases. Such a cotransport mechanism would facilitate transport of sucrose into the cytoplasm of the respective cells, where the GH32 functional domain may then process sucrose into glucose and fructose to provide nourishment for the nematode. Empirical testing of the latter hypothesis will be of particular interest for future studies of root-knot nematode INVs.

Lastly, for the Hoplolaimina *chorismate mutase* (*CM*) gene family, a subtree was generated from the raw phylogenetic tree shown in figure S4, and is presented in figure S8. We observed four clusters of Hoplolaimina *CMs*. Clusters 1 (little support; boostrap of only 57) and 2 were composed of the root-knot nematode *Meloidogyne arenaria* and the false root-knot nematode *N. aberrans*, and exclusively root-knot nematodes, respectively, and thus Hoplolaimina clade A (see fig. 1B). Clusters 3 and 4 contained cyst nematodes and *Ro. reniformis*, and thus Hoplolaimina clade 2B (fig. S8; see fig. 1B for reference). The overall pattern of the clusters suggested that clusters 1, 2 and 3 contain the xenologs of the *Burkholderia*-related *CMs*, while there appeared to be a duplication of *CMs* in Hoplolaimina clade 2B that resulted in the *GLAND16s* in cluster 4. Since there is little support for cluster 1, and cluster 1's grouping with *Burkholderia* was not at all supported, in addition to the finding that the grouping of clusters 2 and 3/4 is not supported, we could not determine which *M. arenaria* paralog (revealing another duplication) is the more likely xenolog.

As mentioned throughout the main text, the relative sizes of GLAND16s are interesting (fig. S8; we could not obtain the complete sequences for the *H. avenae* and *Ro. reniformis* orthologs, indicated by dots in the C-termini), and as mentioned above, the gene forms first appeared in the Hoplolaimina clade B lineage (see fig. 1B). Interestingly, although in a blastp search against the non-redundant (NR) database using the amino acids that flanked the GLAND16 CM domains we did not identify any similar sequences, in a separate search of all nematode genome and transcriptome sequences at Nematode.net we identified significant matches to novel sequences from numerous nematodes (see Methods, and data not shown). This latter finding suggested that the *CM* domain derived from *Burkholderia*-related bacteria was inserted into a nematode gene resulting in *GLAND16s* (fig. S8), somewhat similar to what we observed for the class 3 *Mi-INV* (fig. S7). Another interesting finding, although predicted signal peptides were found at the N-termini of nearly all CM homologs, this was not observed for *N. aberrans*

(fig. S8). Na-CM does not contain a signal peptide, but rather, two TM regions in the N-terminal half of the protein. Given that the shikimate pathway is believed to be absent from animals, and thus nematodes, but present in plants, Na-CM not being secreted into the plant, but rather, likely functioning within the nematode is somewhat paradoxical. It will be interesting to see in future studies what the significances are for the evident neofunctionalizations of both the INVs and CMs in particular Hoplolaimina PPN.
